# Supplementary material for: Reduction of Li+ within a borate anion
Source: Nat Commun. 2024 Mar 22;15:2590. doi: 10.1038/s41467-024-46948-8 (PMC10960030; doi:10.1038/s41467-024-46948-8)
Supplement: Supplementary file 1 — Supplementary Information [file 41467_2024_46948_MOESM1_ESM.pdf]

Supplementary Information

Reduction of Li^+ within A Borate Anion

Haokun Li[1], Jiachen Yao[1], Gan Xu[1], Shek-Man Yiu[1], Chi-Kit Siu[1], Zhen Wang[1], Yung-Kang Peng[1], Yi Xie[2], Ying Wang[2], and Zhenpin Lu[1]*

[1] Department of Chemistry, State Key Laboratory of Marine Pollution, City University of Hong Kong, Kowloon Tong, Hong Kong SAR, P. R. China, E-mail: zhenpilu@cityu.edu.hk

[2] Department of Chemistry, The Chinese University of Hong Kong, Shatin, Hong Kong SAR, P. R. China

Table of Contents

Table of Contents 2
General Information 3
Experimental Section 4
Gas Chromatography 42
Cyclic Voltammetry 47
EPR Spectroscopy 48
Powder XRD Analysis 50
X-ray photoelectron spectroscopy (XPS) 51
ICP-OES Study 52
Crystallographic Details 55
Computational Details 56
Reactions of Scheme 6-8 using naphthalene anion as reducing reagents 61
References 66

General Information

All synthetic experiments and product purifications were conducted using glovebox (Ar) or Schlenk (N_2) techniques (except for preparative thin layer chromatography, PTLC, and column chromatography). Chemicals were purchased from Sigma Aldrich, TCI, Aladdin, Macklin, or Bidepharm and used without further treatment. SPS-purified THF, toluene, and *n*-hexane were dried using 3 Å molecular sieves in the glovebox. Deuterated solvents ($CDCl_3$, C_6D_6) were degassed by freeze-pump-thaw cycles and stored over 3 Å molecular sieves in the glovebox (THF-d_8 was dried using NaK before use, and deuterated solvents for air-stable products were used without further treatment). CO_2 (99.99%) was purchased from Linde Industrial Gases and dried with a combination of a P_2O_5 drying column and 3 Å molecular sieves over one week. The nuclear magnetic resonance (NMR) spectroscopy was facilitated over a Bruker Avance series of spectrometers with frequencies of 300, 400, and 600 MHz. Chemical shifts (δ) are presented in ppm and set relative to the respective solvent's residual proton (^{1}H) signal or the carbon nuclei (^{13}C{^{1}H}).[1] ^{11}B, ^{119}Sn{^{1}H}, ^{31}P{^{1}H} NMR spectra were referenced to external standards, $BF_3{\cdot}OEt_2$, $SnMe_4$ and 85% H_3PO_4, respectively. High-resolution mass spectra were recorded via a Sciex X500R Q-TOF mass spectrometer.

Experimental Section

Synthesis of (bipy)BPh (4):

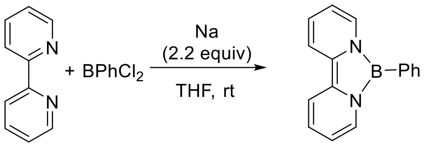

4

67%

To a solution of 2,2'-Bipyridine (1075 mg, 6.89 mmol, 1.0 eq.) in THF (20 mL) was added $BPhCl_2$ (1096 mg, 6.89 mmol, 1.0 eq.). The mixture was stirred at room temperature for 5 min. Then, sodium (348 mg, 15.13 mmol, 2.2 eq.) was added to the reaction mixture at room temperature, and the reaction was stirred at room temperature overnight. Afterward, all the volatile was removed under a high vacuum, and toluene (50 mL) was added to extract the target product. After filtration, the toluene phase was reduced under high vacuum, affording compound **4** as a red solid (1132 mg, 67%). Compound **4** was pure at this stage, and no further purification was needed.

^{1}H NMR (400 MHz, C_6D_6, 298K): δ [ppm] = 7.64 (d, $J_{H\text{-}H}$ = 7.2 Hz, 2H), 7.49 (d, $J_{H\text{-}H}$ = 6.1 Hz, 2H), 7.31-7.23 (m, 3H), 7.19 (d, $J_{H\text{-}H}$ = 9.1 Hz, 2H), 6.18 (dd, $J_{H\text{-}H}$ = 9.3, 6.0 Hz, 2H), 5.92 (t, $J_{H\text{-}H}$ = 6.6 Hz, 2H).

^{11}B NMR (128 MHz, C_6D_6, 298K): δ [ppm] = 21.56.

^{13}C{^{1}H} NMR (100 MHz, C_6D_6, 298K): δ [ppm] = 133.84, 129.33, 128.71, 127.25, 118.99, 118.55, 114.77, 110.93.

HRMS (m/z): Calc. For **[4]** ($C_{16}H_{13}BN_2$): 244.1172 Found: 244.1169.

Synthesis of [Li(THF)$_4$][(bipy)BPh$_2$] (5):

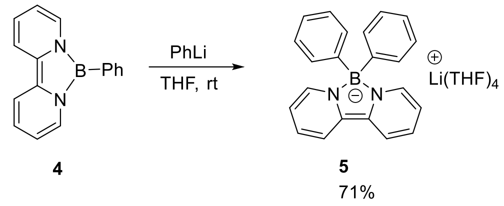

To a solution of **[4]** (300 mg, 1.23 mmol, 1.0 eq.) in THF (10 mL) was added PhLi (103 mg, 1.23 mmol, 1.0 eq.) at room temperature. The reaction mixture was stirred at room temperature overnight. Afterward, the mixture was filtered. The filtrate was collected and reduced under a high vacuum, affording **5** as a black solid (540 mg, 71%).

Single crystals suitable for X-ray crystal structure analysis were obtained from vapor diffusion of n-hexane to THF solution at room temperature.

^{1}H NMR (400 MHz, THF-d$_8$, 298K): δ [ppm] = 7.42 (d, $J_{H\text{-}H}$ = 7.3 Hz, 4H), 7.03 (t, $J_{H\text{-}H}$ = 7.3 Hz, 4H), 6.88 (t, $J_{H\text{-}H}$ = 7.3 Hz, 2H), 5.68 (d, $J_{H\text{-}H}$ = 6.7 Hz, 2H), 5.11 (d, $J_{H\text{-}H}$ = 9.4 Hz, 2H), 4.64 (dd, $J_{H\text{-}H}$ = 9.5, 5.3 Hz, 2H), 3.82 (t, $J_{H\text{-}H}$ = 6.1 Hz, 2H).

^{11}B NMR (128 MHz, THF-d$_8$, 298K): δ [ppm] = 2.79.

^{13}C{^{1}H} NMR (100 MHz, THF-d$_8$, 298K): δ [ppm] = 140.43, 133.96, 126.66, 124.27, 119.68, 117.64, 116.24, 94.50.

HRMS (m/z): Calc. For **[5-Li(THF)$_4$]** ($C_{22}H_{18}BN_2$): 321.1563 Found: 321.1548

Synthesis of [K(THF)$_2$][(bipy)BPh$_2$] (5'):

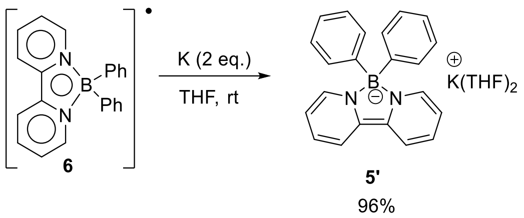

To a solution of **[6]** (367 mg, 1.14 mmol, 1.0 eq.) in THF (10 mL) was added potassium (89 mg, 2.28 mmol, 2.0 eq.) at room temperature. The reaction mixture was stirred at room temperature overnight. Afterward, the mixture was filtered. The filtrate was collected and reduced under a high vacuum, affording compound **5'** as a black solid (552 mg, 96%).

^{1}H NMR (400 MHz, THF-d$_8$, 298K): δ [ppm] = 7.42 (d, $J_{H\text{-}H}$ = 7.2 Hz, 4H), 7.09 (t, $J_{H\text{-}H}$ = 7.3 Hz, 4H), 6.95 (t, $J_{H\text{-}H}$ = 7.3 Hz, 2H), 5.70 (d, $J_{H\text{-}H}$ = 6.8 Hz, 2H), 5.20 (d, $J_{H\text{-}H}$ = 9.5 Hz, 2H), 4.73 (dd, $J_{H\text{-}H}$ = 9.5, 5.3 Hz, 2H), 3.93 (t, $J_{H\text{-}H}$ = 6.1 Hz, 2H).

^{11}B NMR (128 MHz, THF-d$_8$, 298K): δ [ppm] = 2.73.

^{13}C{^{1}H} NMR (100 MHz, THF-d$_8$, 298K): δ [ppm] = 139.66, 133.32, 126.97, 124.75, 118.84, 117.29, 116.54, 95.25.

HRMS (m/z): Calc. For **[5'-K(THF)$_2$]** ($C_{22}H_{18}BN_2$): 321.1563 Found: 321.1555.

Synthesis of [(bipy)BPh_2] (6):

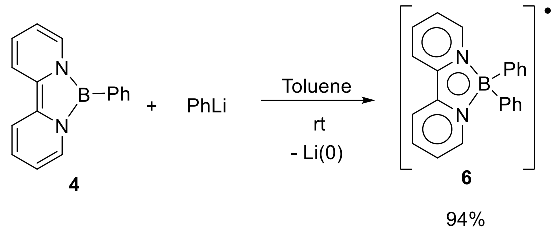

From 4: To a solution of **[4]** (210 mg, 0.86 mmol, 1.0 eq.) in toluene (10 mL) was added PhLi (108 mg, 1.29 mmol, 1.5 eq.) at room temperature. The reaction mixture was stirred at room temperature overnight. Afterward, the mixture was filtered. The filtrate was collected and reduced under a high vacuum, affording compound **6** as a deep green solid (260 mg, 94%).

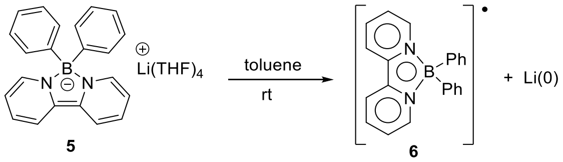

From 5: Toluene (10mL) was added to **[5]** (255 mg, 0.41 mmol) in the glovebox and stirred at room temperature overnight. Afterward, the mixture was filtered. The filtrate was collected and reduced under a high vacuum, affording compound **6** as deep green powder. (113 mg, 85%)

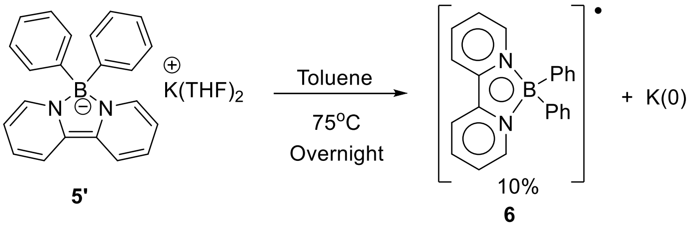

From 5': Compound (**5'**) (35 mg, 0.070 mmol) was suspended in toluene (10 mL) at room temperature. The mixture was heated without stirring at 75°C overnight. The color of the toluene solution turned dark green. After filtration, the toluene solution was collected, and all the volatile was removed under a high vacuum, affording compound **6** (2.3 mg, 10%).

Single crystals suitable for X-ray crystal structure analysis were obtained from the slow evaporation of a toluene solution at room temperature or recrystallization at -35°C in THF.

^{1}H NMR (400 MHz, C_6D_6, 298K): silence.

^{11}B NMR (128 MHz, C_6D_6, 298K): silence.

g-factor (toluene, 298K): 2.0036

HRMS (m/z): Calc. For **[6]** ($C_{22}H_{18}BN_2$): 321.1563 Found: 321.1552.

Control Experiment:

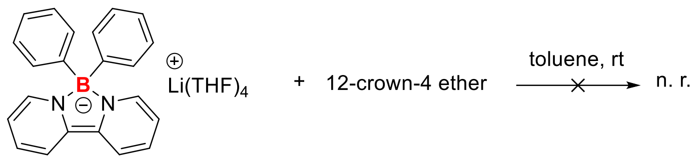

5

12-crown-4 ether (6 mg, 0.035 mmol, 2.2 eq.) was solved in toluene (1 mL) and added to **[5]** (10 mg, 0.016 mmol) in a J. Young's tube. The solution was slightly red, and many black powders remained insoluble. The solution was EPR silence. (Supplementary Figure38)

Synthesis of [(bipy)BPh$_2$][CuCl$_2$] (7):

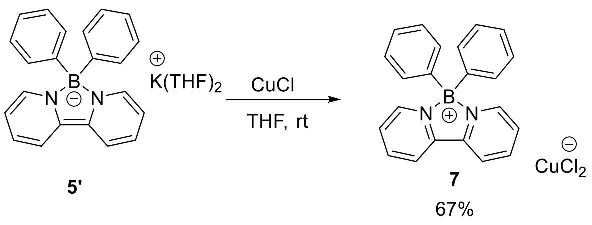

From 5': To THF solution (10 mL) of **[5']** (297 mg, 0.59 mmol, 1.0 eq.) was added CuCl (175 mg, 1.77 mmol, 3.0eq.). The reaction mixture was stirred at room temperature overnight. Afterward, the solution was concentrated to about 2 mL, and n-hexane (5 mL) was added. Red solids precipitated after storage at -35°C overnight. After filtration, the solids were dried over high vacuum, affording compound **7** (180 mg, 67%).

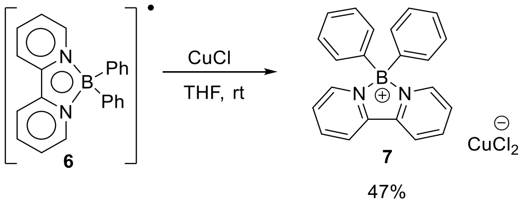

From 6: To THF solution (4 mL) of **[6]** (50 mg, 0.15 mmol, 1.0 eq.) was added CuCl (154 mg, 1.55 mmol, 10 eq.). The reaction was stirred at room temperature overnight. Then, excess CuCl was removed via filtration. All the volatile was removed under a fine vacuum, and **7** was obtained as a red powder (33 mg, 47%).

Single crystals suitable for X-ray crystal structure analysis were obtained from recrystallization at -35°C in the mixture solvent of DCM/n-hexane/THF.

^{1}H NMR (600 MHz, CDCl$_3$, 298K): δ [ppm] = 9.51 (d, J_{H-H} = 8.1 Hz, 2H), 8.79 (t, J_{H-H} = 8.0 Hz, 2H), 8.64 (d, J_{H-H} = 5.7 Hz, 2H), 8.02 (t, J_{H-H} = 6.8 Hz, 2H), 7.38 – 7.31 (m, 6H), 7.13 (d, J_{H-H} = 7.0 Hz, 4H).

^{11}B NMR (128 MHz, CDCl$_3$, 298K): δ [ppm] = 9.75.

^{13}C{^{1}H} NMR (150 MHz, CDCl$_3$, 298K): δ [ppm] = 145.68, 143.40, 132.69, 129.33, 129.10, 128.91, 125.24.

HRMS (m/z): Calc. For **[7-CuCl$_2$]** ($C_{22}H_{18}BN_2$): 321.1563 Found: 321.1557.

P-P coupling reactions:

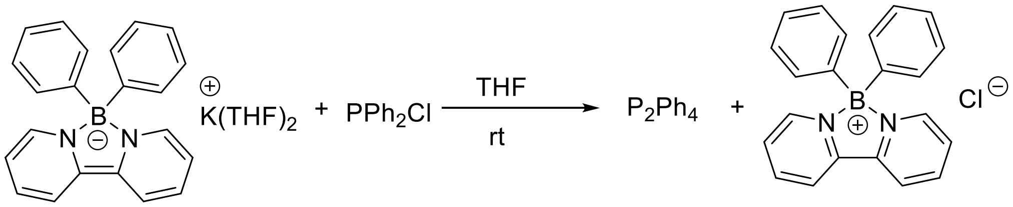

5'   8

71%   80%

From 5': To the THF solution (5 mL) of **[5']** (100 mg, 0.20 mmol, 1.0 eq.) was added PPh_2Cl (90 mg, 0.40 mmol, 2.0 eq.). The reaction was stirred at room temperature overnight, and all the volatile was removed under a high vacuum afterward. The residue was extracted by n-hexane (2×6 mL). P_2Ph_4 was in the hexane solution and obtained after being dried under a high vacuum, giving a yellow powder (54 mg, 71%). The residue was dried under high vacuum as **8** (59 mg, 80%).

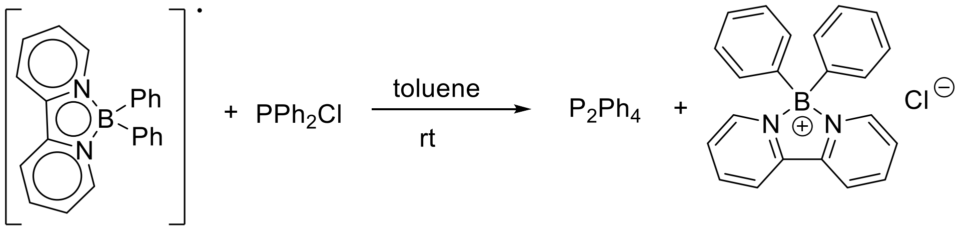

6   8

86%   96%

From 6: To the toluene solution (5 mL) of **[6]** (50 mg, 0.16 mmol, 1.0 eq.) was added PPh_2Cl (35 mg, 0.16 mmol, 1.0 eq.). The reaction was stirred at room temperature overnight. All the volatile was removed under a high vacuum afterward. The residue was extracted by n-hexane (3×3 mL). P_2Ph_4 was in the hexane solution and obtained after being dried under a high vacuum, giving a yellow powder (25 mg, 86%). The residue was dried under high vacuum as **8** (55 mg, 96%).

P_2Ph_4:^{1}H NMR (400 MHz, C_6D_6, 298K): δ [ppm] = 7.58 – 7.52 (m, 4H), 6.97 – 6.91 (m, 6H).

^{31}P{^{1}H} NMR (161 MHz, C_6D_6, 298K): δ [ppm] = -15.01. The spectroscopic data are in accordance with the reported results.[2]

[8]: ^{1}H NMR (400 MHz, $CDCl_3$, 298K): δ [ppm] = 10.72 (d, $J_{H\text{-}H}$ = 8.3 Hz, 2H), 8.71 (t, $J_{H\text{-}H}$ = 8.1 Hz, 2H), 8.57 (d, $J_{H\text{-}H}$ = 5.6 Hz, 2H), 7.95 (t, $J_{H\text{-}H}$ = 6.8 Hz, 2H), 7.40 – 7.29 (m, 6H), 7.08 (d, $J_{H\text{-}H}$ = 7.0 Hz, 4H).

^{11}B NMR (128 MHz, $CDCl_3$, 298K): δ [ppm] = 9.16

^{13}C{^{1}H} NMR (100 MHz, $CDCl_3$, 298K): δ [ppm] = 145.70, 142.63, 132.55, 129.10, 128.94, 128.64, 127.30.

Sn-Sn coupling reactions:

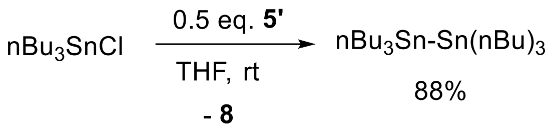

To the THF solution (3 mL) of **[5']** (50 mg, 0.10 mmol, 1 eq.) was added nBu_3SnCl (64 mg, 0.20 mmol, 2.0 eq.). The reaction was stirred at room temperature overnight, and all the volatile was removed under a high vacuum afterward. The residue was extracted by n-hexane, and n-Bu_6Sn_2 was obtained after removing all the volatiles under a high vacuum (50 mg, 88%).

nBu_6Sn_2: ^{1}H NMR (400 MHz, C_6D_6, 298K): δ [ppm] = 1.80 – 1.58 (m, 12H), 1.47 – 1.36 (m, 12H), 1.26 – 1.07 (m, 12H), 0.98 (t, $J_{H\text{-}H}$ = 7.3 Hz, 18H).

^{13}C{^{1}H} NMR (100 MHz, C_6D_6, 298K): δ [ppm] = 29.87, 27.74, 13.78, 10.23.

^{119}Sn{^{1}H} NMR (149 MHz, C_6D_6, 298K): δ [ppm] = -83.72. The spectroscopic data are in accordance with those reported in the literature.[3]

Se-Se coupling reactions:

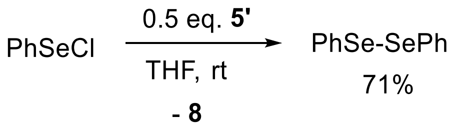

PhSeCl (38 mg, 0.20 mmol, 2.0 eq.) was added to the THF solution (3 mL) of **[5']** (50 mg, 0.10 mmol, 1.0 eq.). The reaction was stirred at room temperature overnight, and all the volatile was removed under a high vacuum afterward. Ph_2Se_2 was successfully isolated using PTLC (n-hexane) and obtained as a yellow powder (22 mg, 71%).

Ph_2Se_2: ^{1}H NMR (400 MHz, $CDCl_3$, 298K): δ [ppm] = 7.66 – 7.57 (m, 4H), 7.34-7.21 (m, 6H). The spectroscopic data are in accordance with those reported in the literature.[4]

Ge-Ge coupling reactions:

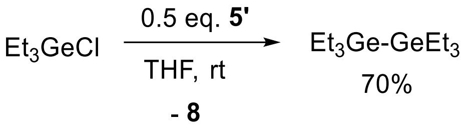

A THF solution (1 mL) of Et_3GeCl (38 mg, 0.20 mmol, 2.0 eq.) was added to **[5’]** (50 mg, 0.10 mmol, 1.0 eq.) in THF (3 mL). The reaction was stirred at room temperature overnight, and all the volatile was removed under a high vacuum afterward. Et_6Ge_2 was purified through column chromatography using n-hexane as an eluent and obtained as a colorless oil (11 mg, 70%).

Et_6Ge_2: ^{1}H NMR (400 MHz, $CDCl_3$, 298K): δ [ppm] = 1.05 (t, $J_{H\text{-}H}$ = 7.7 Hz, 18H), 0.86 (q, $J_{H\text{-}H}$ = 7.6 Hz, 12H).

^{13}C{^{1}H} NMR (100 MHz, $CDCl_3$, 298K): δ [ppm] = 10.08, 5.44. The spectroscopic data are in accordance with the results reported in the literature.[5]

H-H coupling reactions:

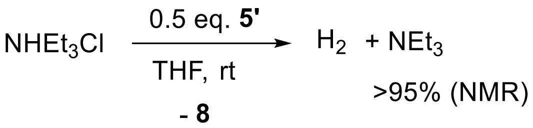

Compound **[5’]** (50 mg, 0.10 mmol, 1.0 eq.) and $NHEt_3Cl$ (27 mg, 0.20 mmol, 2.0 eq.) were mixed in THF-d_8 (0.55 mL) at room temperature. After 12 hours, the clean generation of NEt_3 was observed in ^{1}H NMR (Supplementary Figure 24) [1], and the formation of H_2 was confirmed by GC spectra (Supplementary Figure 28).

Reductive Pyridine couplings:

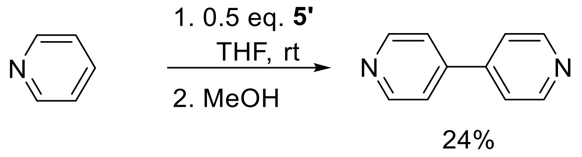

To the THF solution (3 mL) of **[5']** (100 mg, 0.20 mmol, 1.0 eq.) was added pyridine (32 mg, 0.40 mmol, 2.0 eq.). The reaction was stirred at room temperature for 5 days or 60°C overnight. Drops of MeOH were added to quench the reaction. 4,4'-bipyridine was isolated using PTLC (EA: DCM=2:1, 1%NEt_3) and obtained as a yellow powder (7.6 mg, 24%).

4,4'-bipyridine: ^{1}**H NMR** (400 MHz, $CDCl_3$, 298K): δ [ppm] = 8.74 (dd, J_{H-H} = 1.7 Hz, 4H), 7.53 (dd, J_{H-H} = 1.7 Hz, 4H). The spectroscopic data are in accordance with those reported in the literature.[6]

The reduction of acridine:

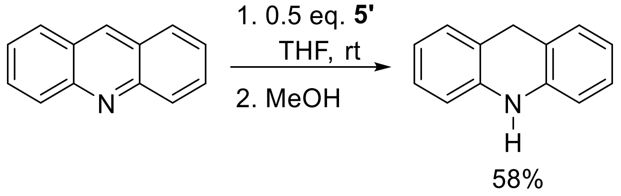

To the solid mixture of **[5']** (50 mg, 0.10 mmol, 1.0 eq.) and acridine (36 mg, 0.20 mmol, 2.0 eq.) was added THF (5 mL). The reaction was stirred at room temperature overnight. Drops of MeOH were added to quench the reaction. Acridan was isolated using PTLC (EA: hex=1:5) and obtained as a white powder (21 mg, 58%).

Acridan: ^{1}**H NMR** (400 MHz, $CDCl_3$, 298K): δ [ppm] = 7.12 - 7.05 (m, 4H), 6.85 (td, J_{H-H} = 7.4 Hz, 1.2 Hz, 2H), 6.67 (dd, J_{H-H} = 7.8 Hz, 1.2 Hz, 2H), 5.96 (s, 1H), 4.06 (s, 2H). The spectroscopic data are in accordance with the results reported in the literature.[6]

CO_2 reactions:

5': The THF solution (10 mL) of **[5']** (50 mg, 0.10 mmol) was degassed in a 250 mL Schlenk tube. An atmosphere of CO_2 was then introduced to the reaction mixture. The reaction was stirred at room temperature overnight. A lot of insoluble solids precipitated. The generation of CO was confirmed by in-situ GC-spectra (Supplementary Figure31). The solids were filtered and washed with THF (1 mL, thrice). The solids were soluble in D_2O. **$^{13}C\{^1H\}$ NMR** (100 MHz, D_2O, 298K) spectroscopy was subsequently deployed, where a signal at δ (ppm) = 163.59 was detected (Supplementary Figure27). However, this signal disappeared after the addition of an excess of HCl (2M in dioxane).

Catalytic fashion: The THF solution (10 mL) of **[5']** (39 mg, 0.077 mmol, 1.0 eq.) and K (122 mg, 3.12 mmol, 40 eq.) was degassed in a 100mL Schlenk tube. A controlled CO_2 atmosphere at 1 atm was introduced (100 mL, about 4 mmol). After being stirred at room temperature for three days, the solution underwent a transformation whereby the potassium was no longer visible, and a lot of insoluble solids precipitated. All the volatiles were reduced from the setup, and the resultant organic products were meticulously rinsed using acetone. After removing the acetone, K_2CO_3 was obtained as a grey powder (152 mg, 70%). **$^{13}C\{^1H\}$ NMR** (100 MHz, D_2O, 298K): δ [ppm] = 168.33. The spectroscopic data are in accordance with that of commercial K_2CO_3.

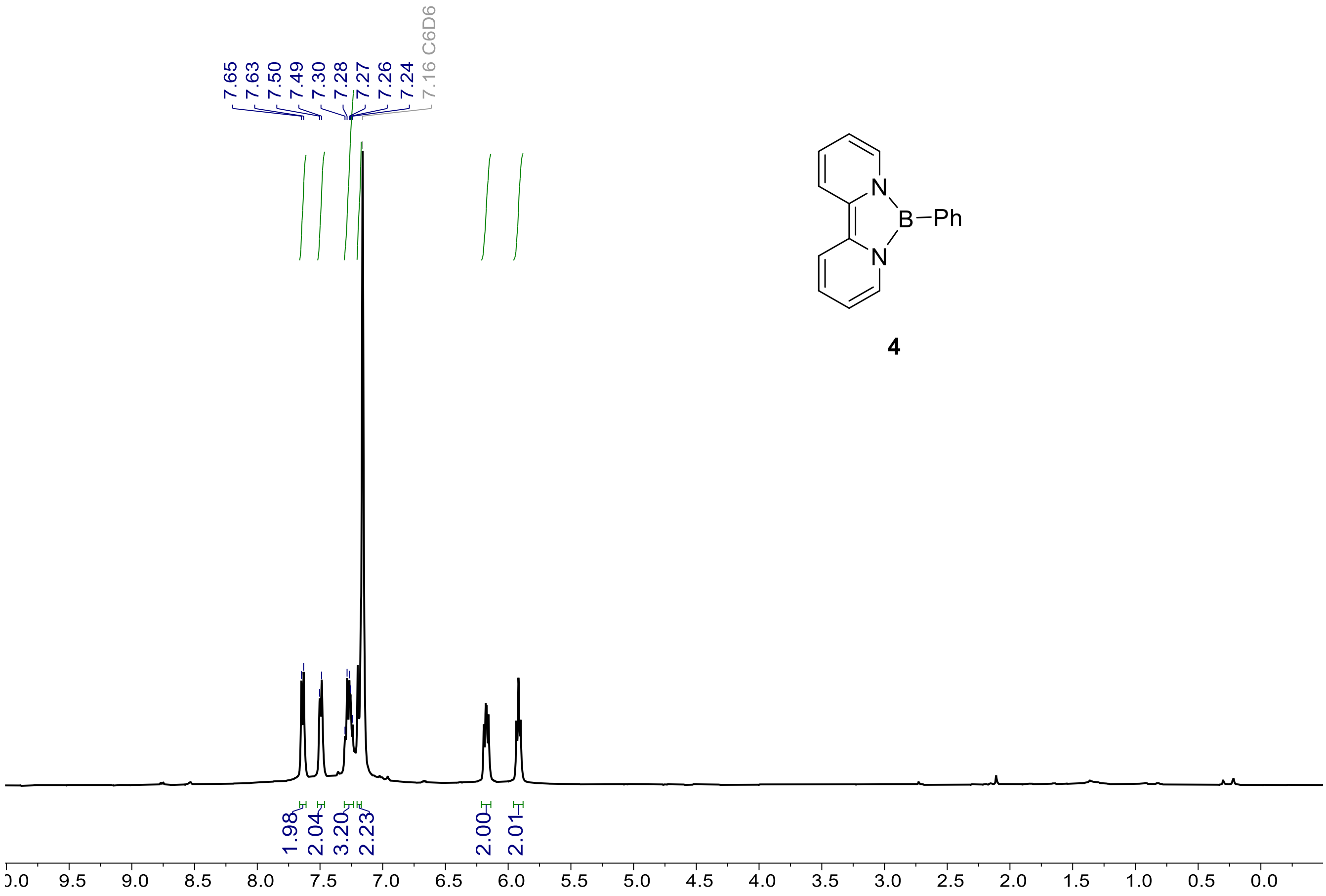

Supplementary Figure 1. ^{1}H NMR of **4** in C_6D_6

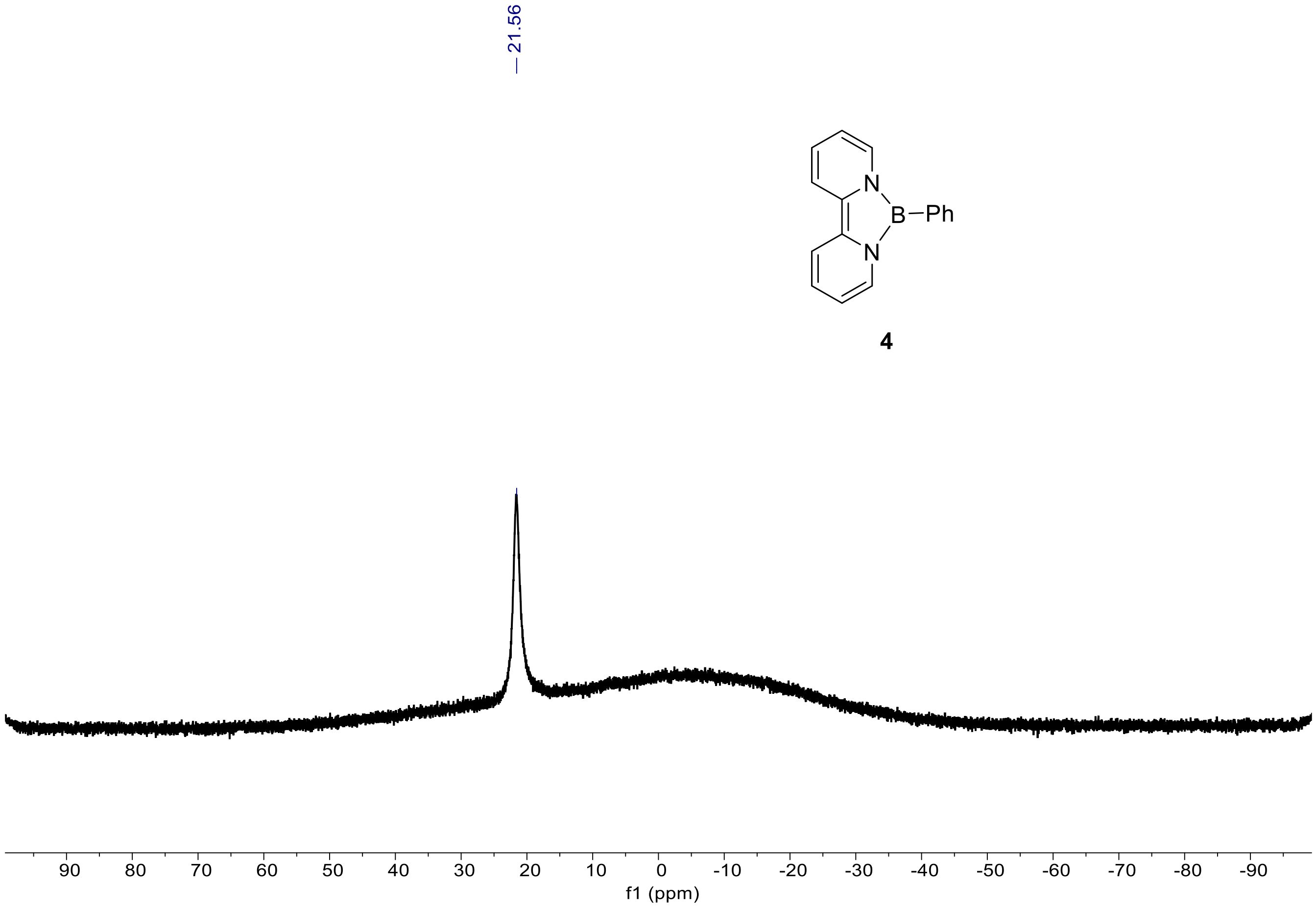

Supplementary Figure 2. ^{11}B NMR of **4** in C_6D_6

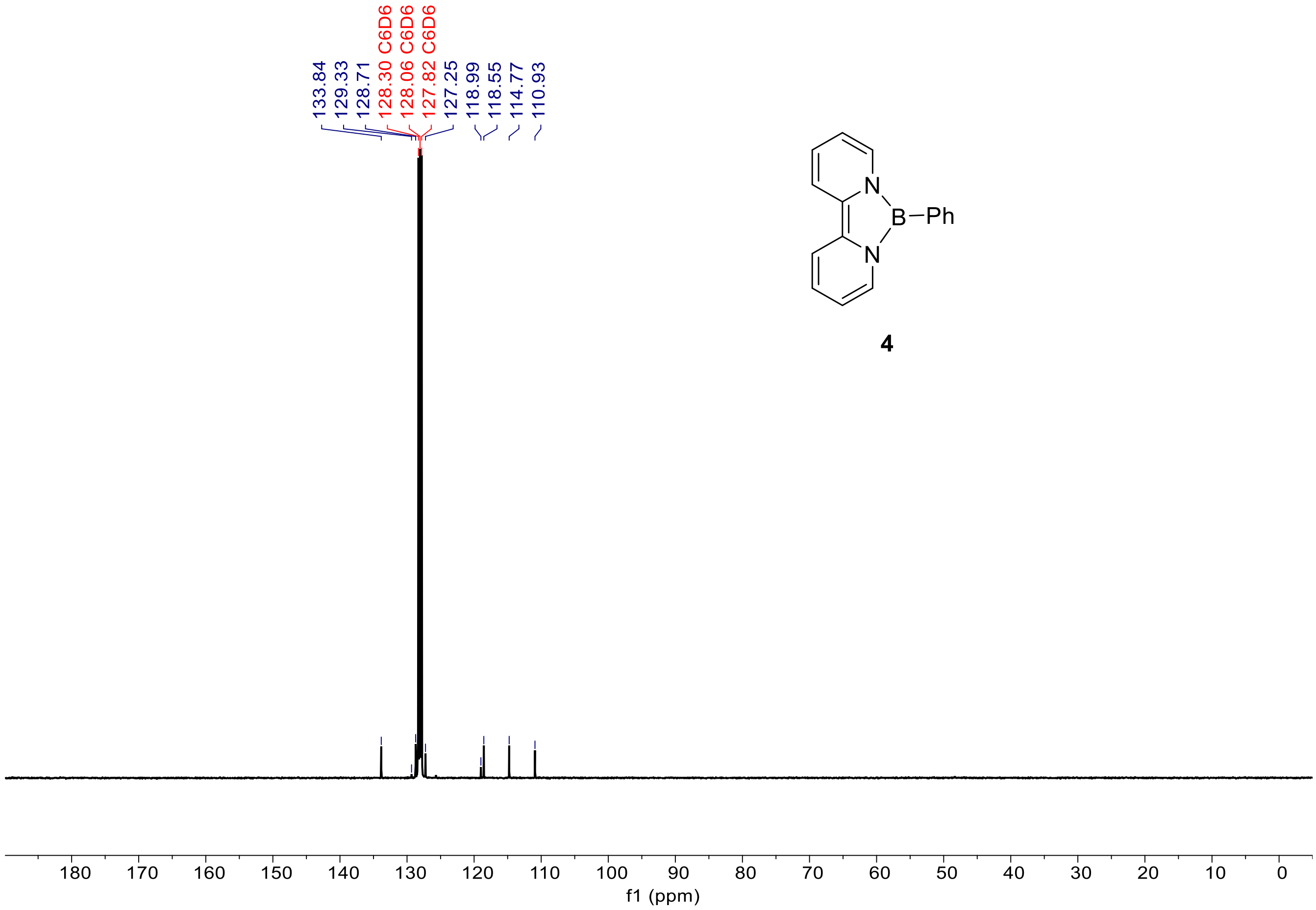

Supplementary Figure 3. $^{13}C\{^1H\}$ NMR of **4** in C_6D_6

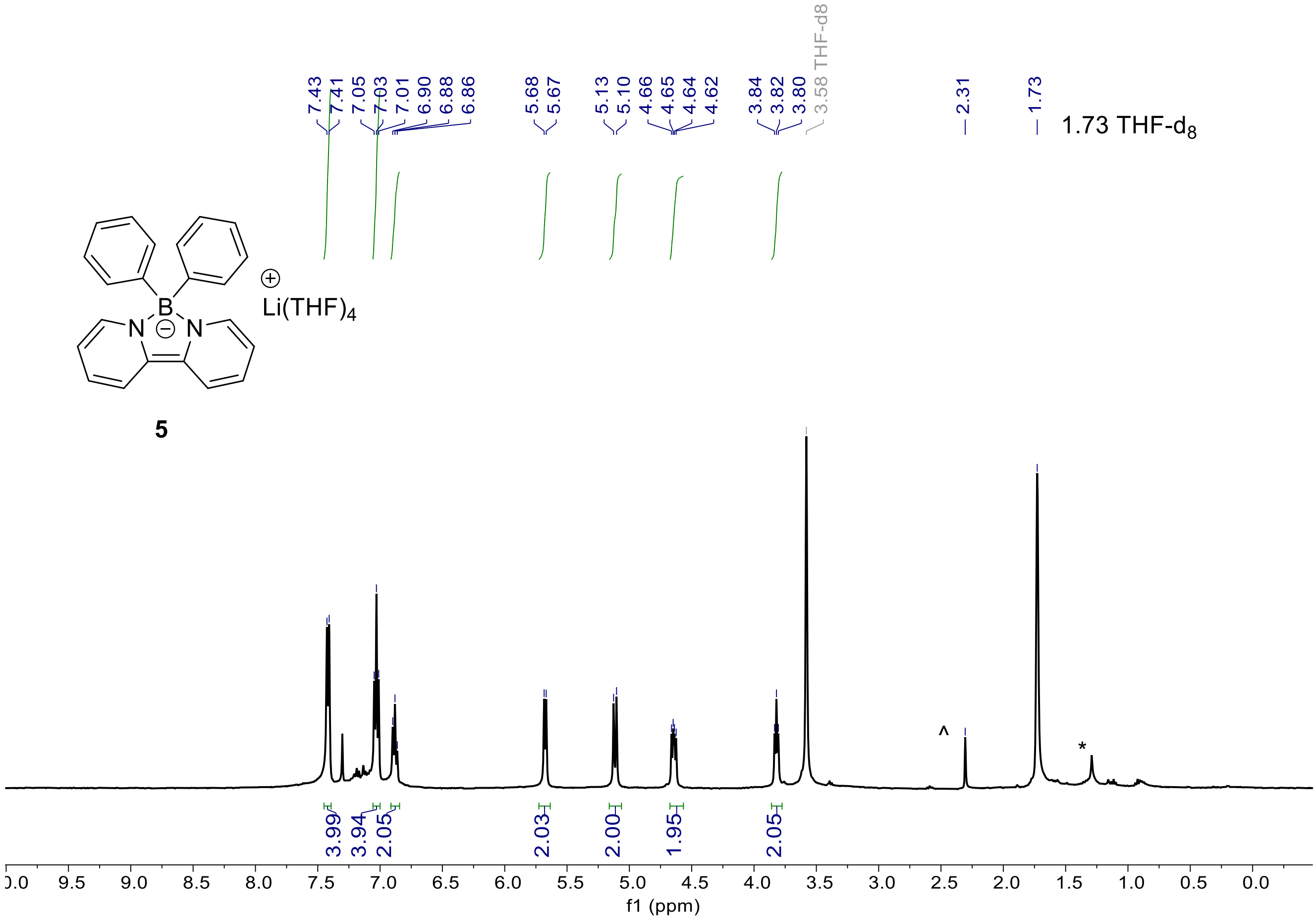

Supplementary Figure 4. ^{1}H NMR of **5** in THF-d8 (*: H grease; ^: toluene)

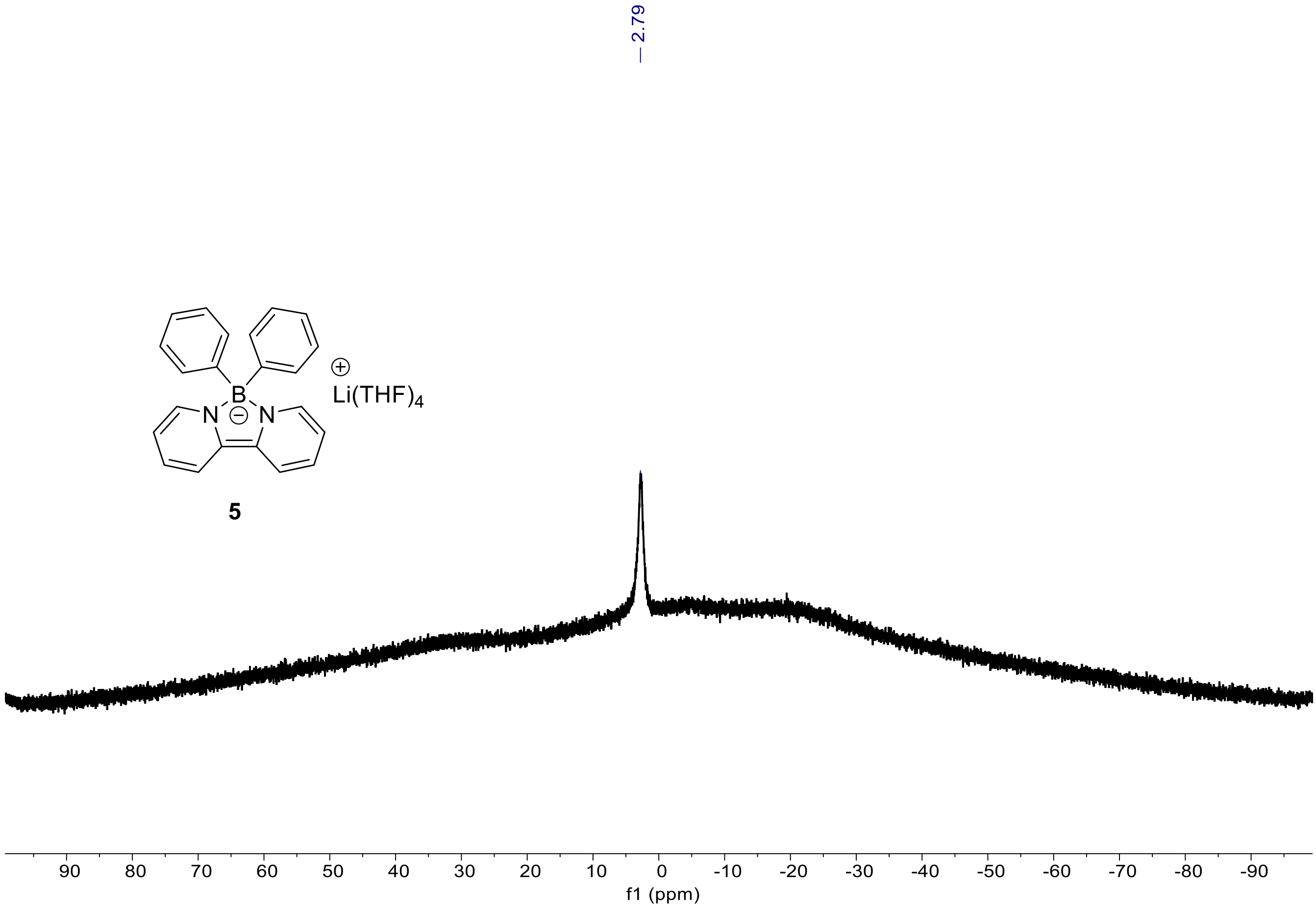

Supplementary Figure 5. ^{11}B NMR of **5** in THF-d_8

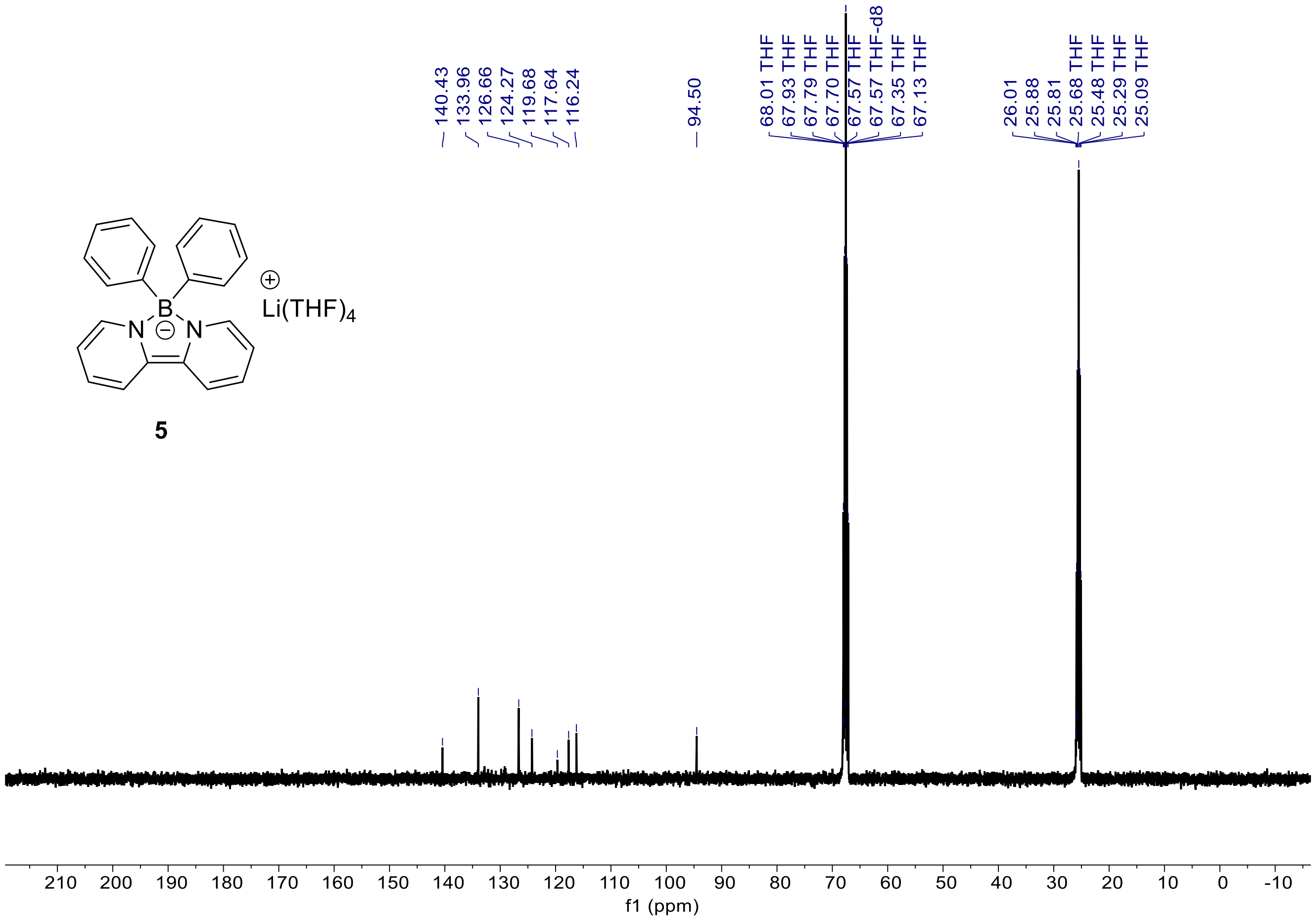

Supplementary Figure 6. $^{13}C\{^{1}H\}$ NMR of **5** in THF-d8

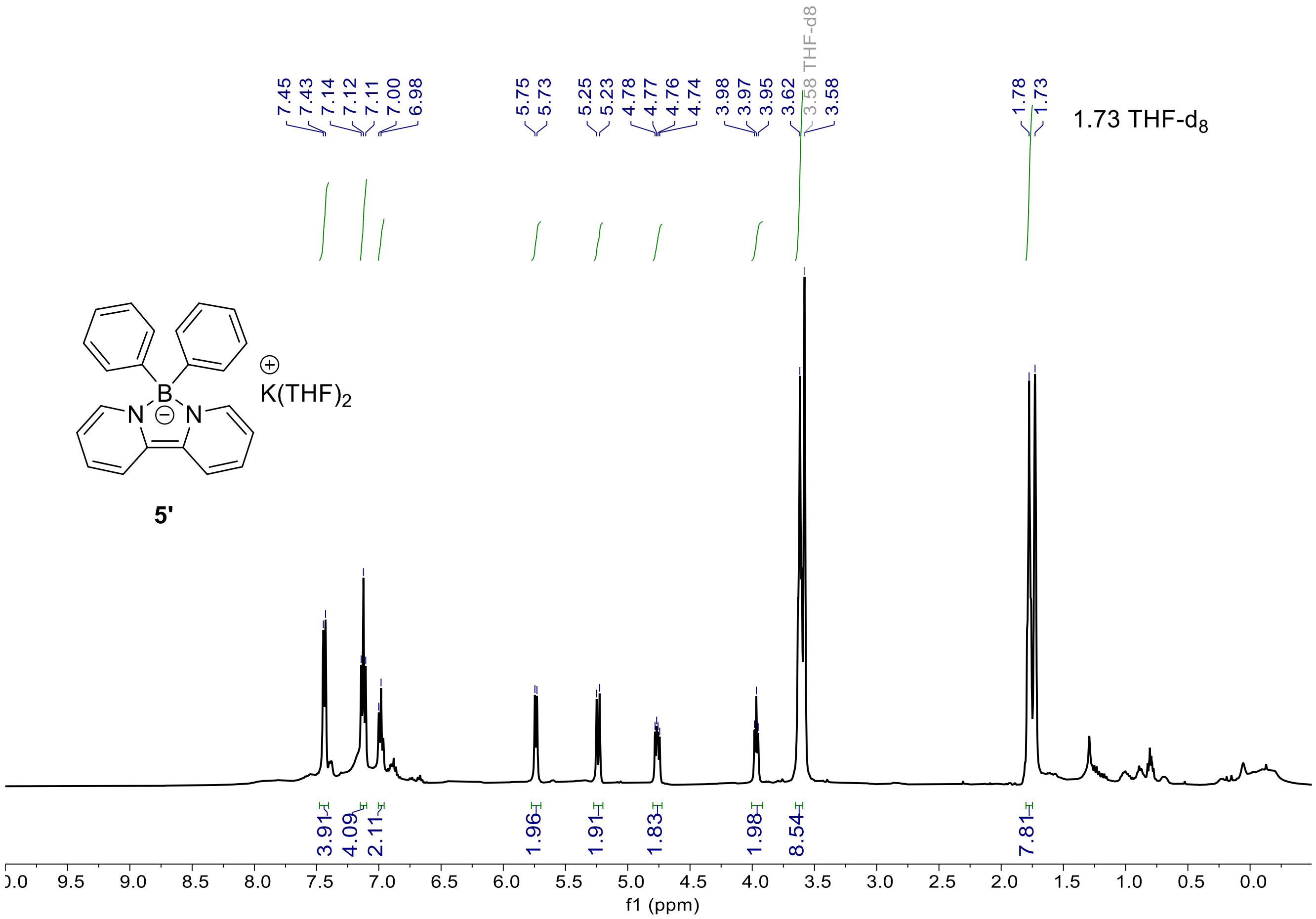

Supplementary Figure 7. ^{1}H NMR of **5'** in THF-d_8

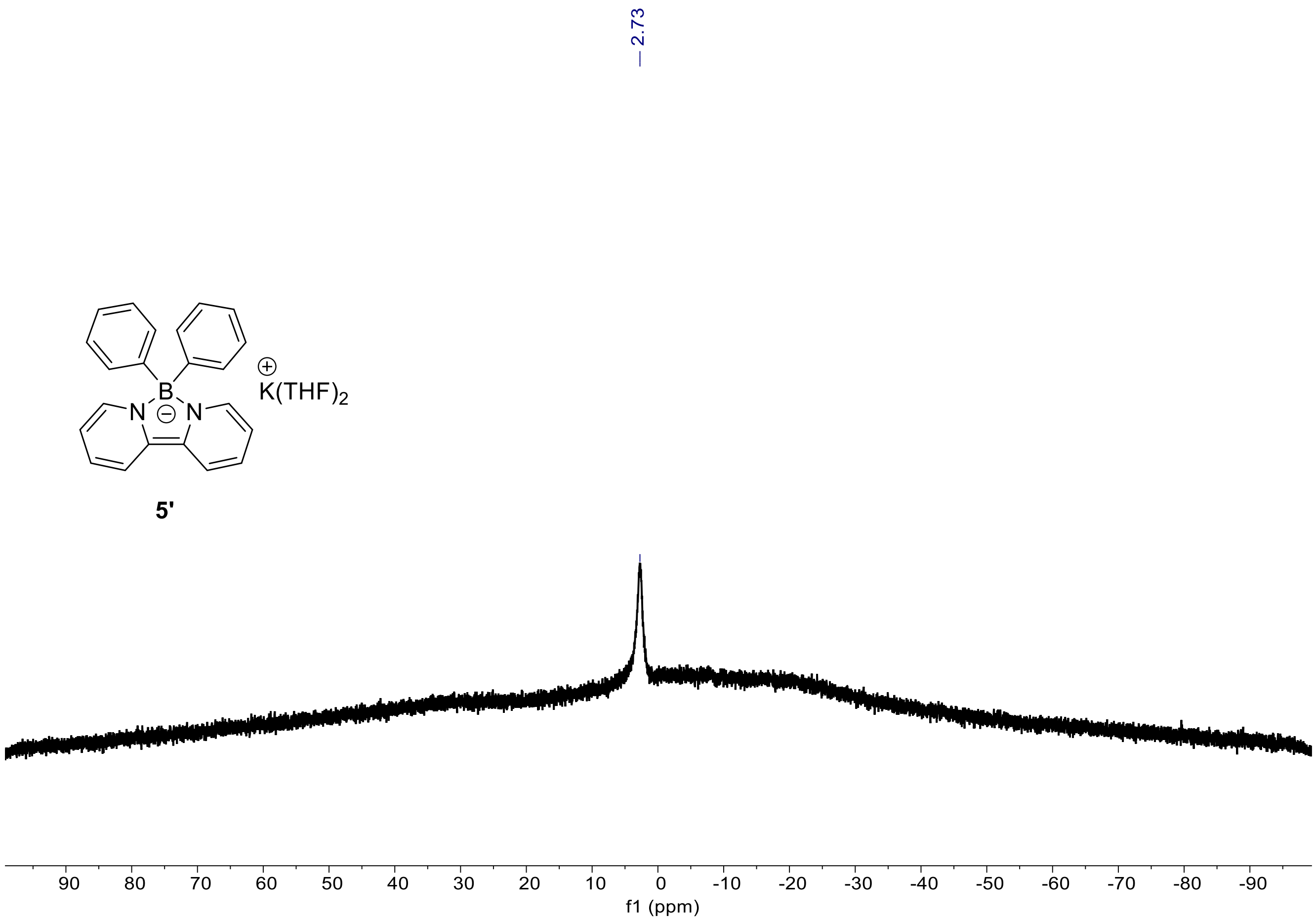

Supplementary Figure 8. ^{11}B NMR of **5’** in THF-d_8

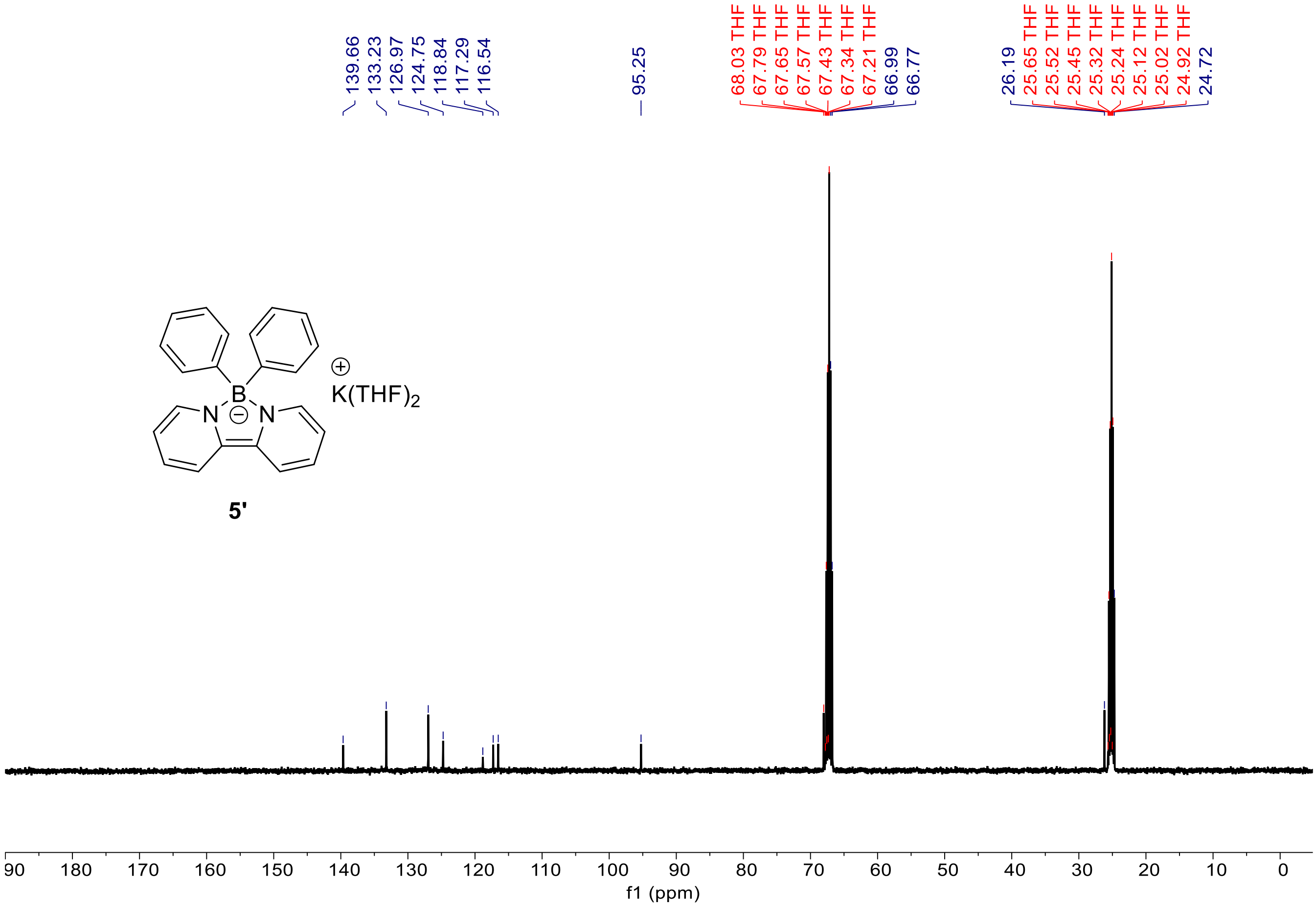

Supplementary Figure 9. $^{13}C\{^{1}H\}$ NMR of **5'** in THF-d8

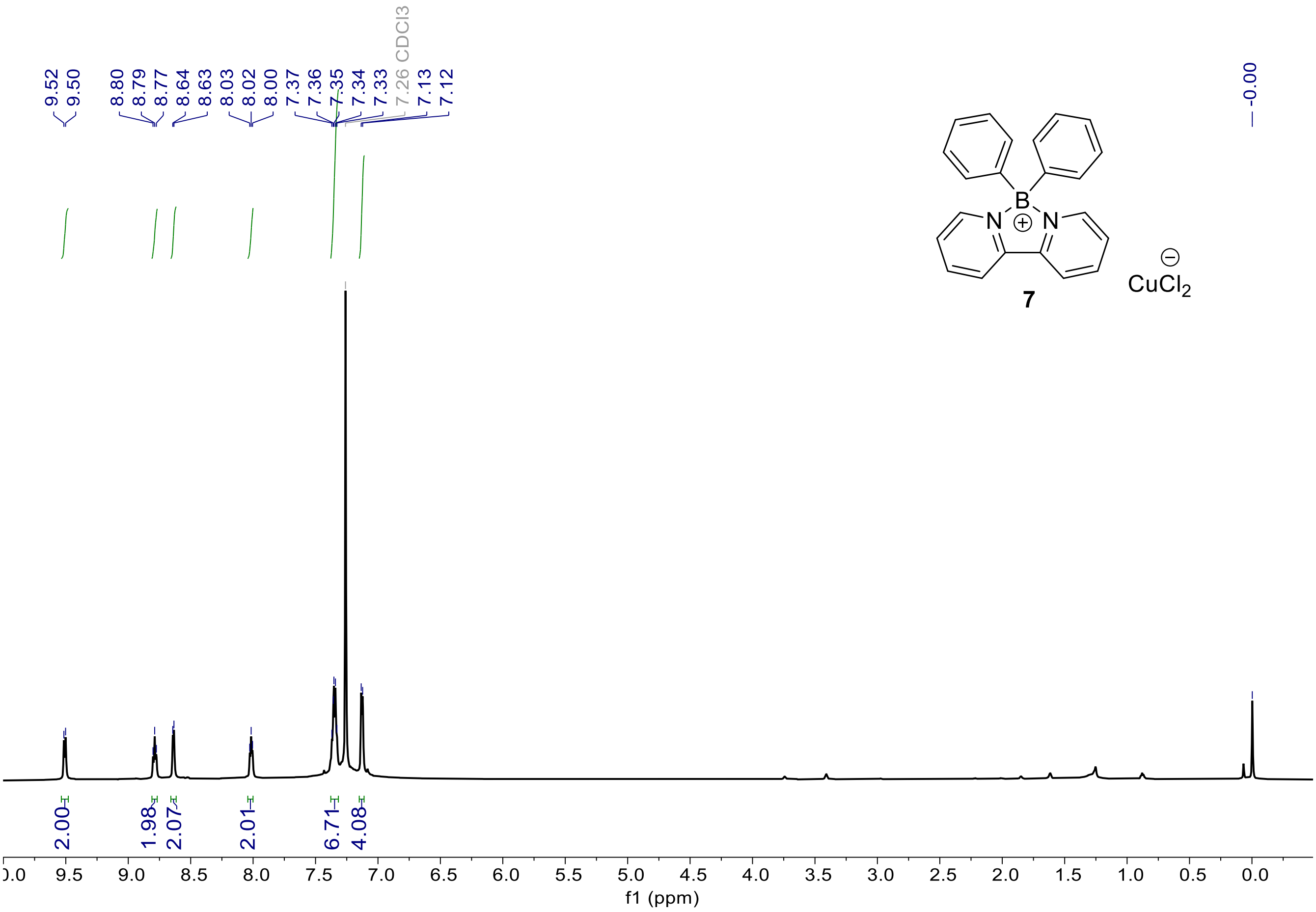

Supplementary Figure 10. ^{1}H NMR of **7** in $CDCl_3$

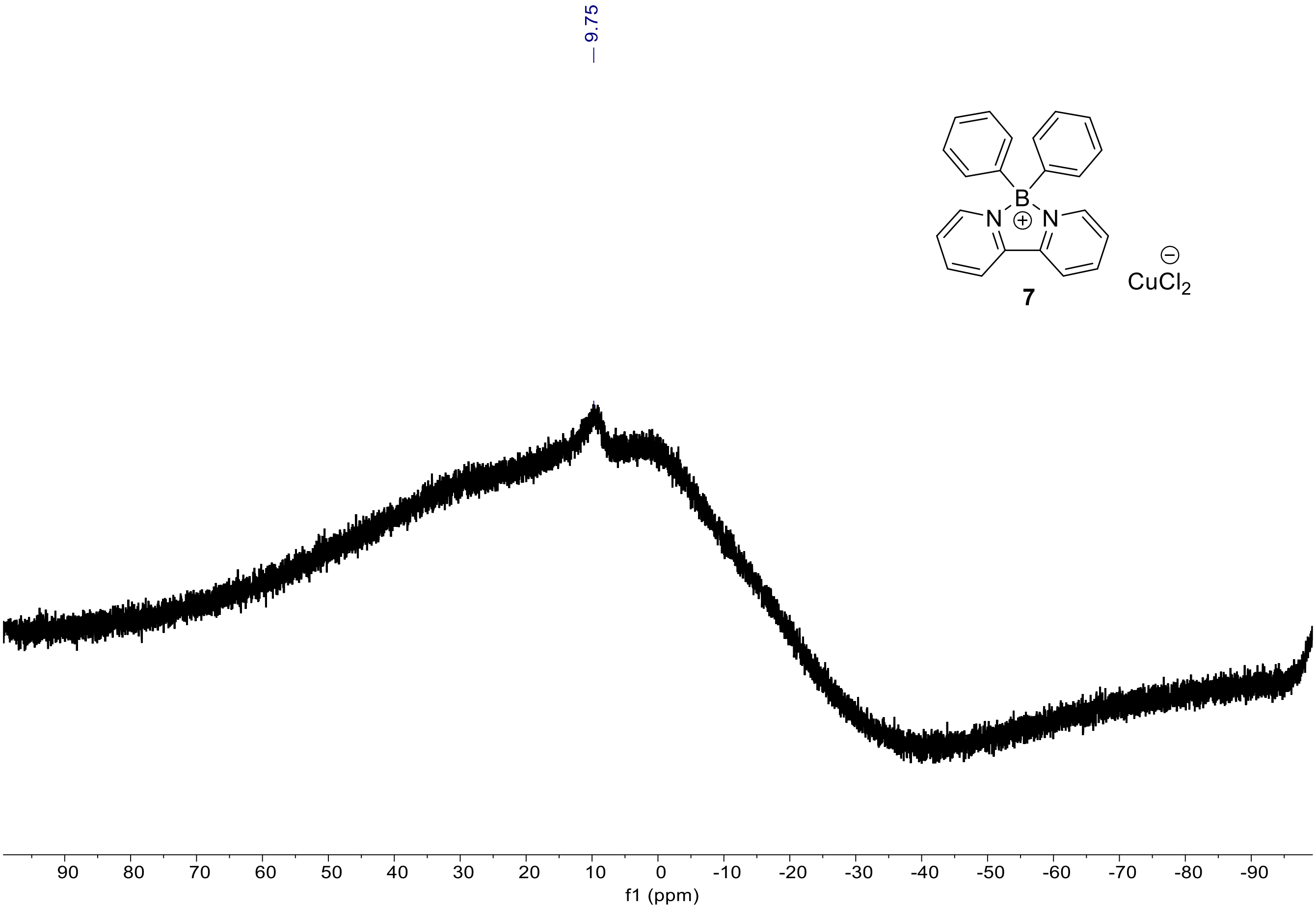

Supplementary Figure 11. ^{11}B NMR of **7** in $CDCl_3$

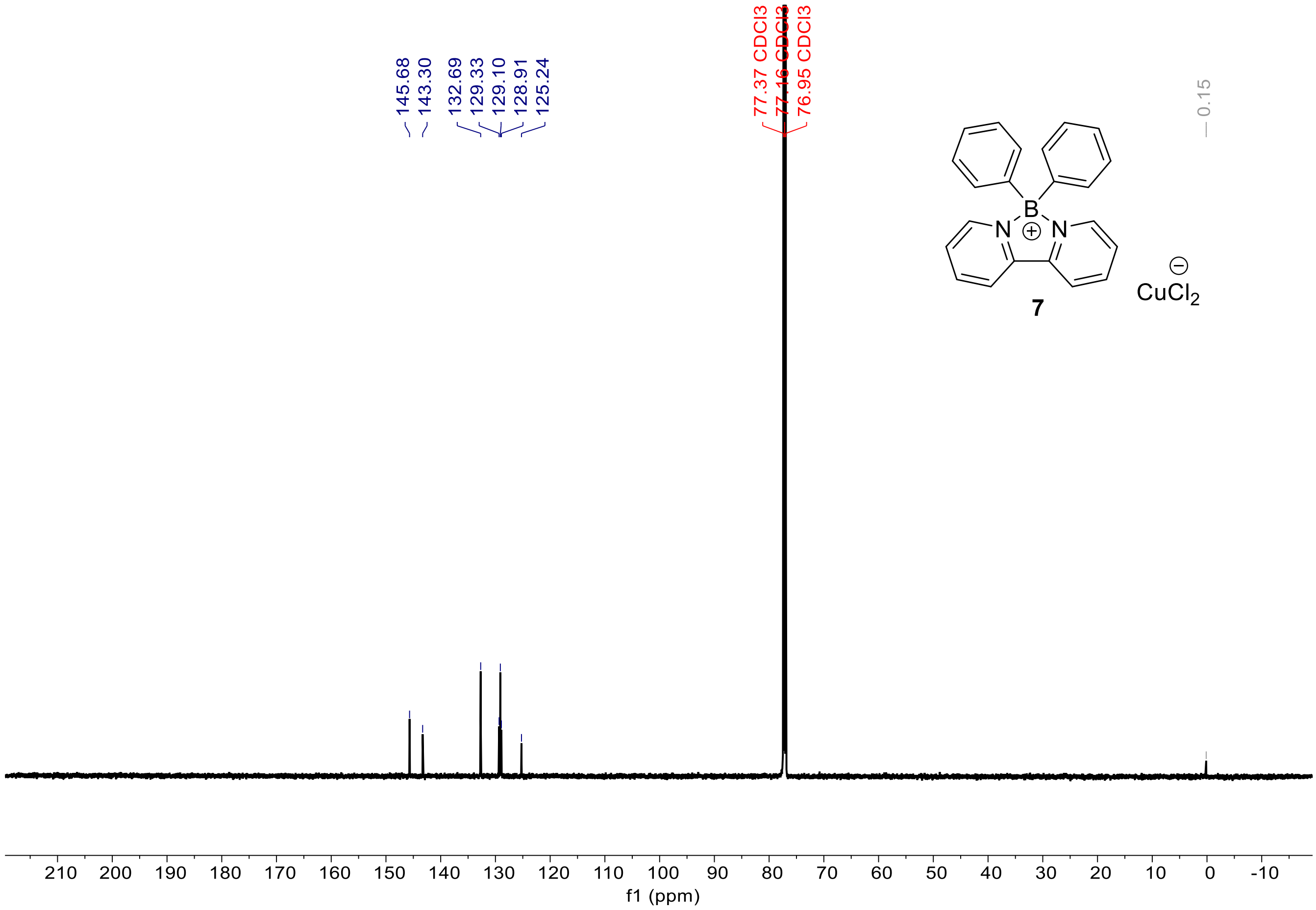

Supplementary Figure 12. $^{13}C\{^1H\}$ NMR of **7** in $CDCl_3$

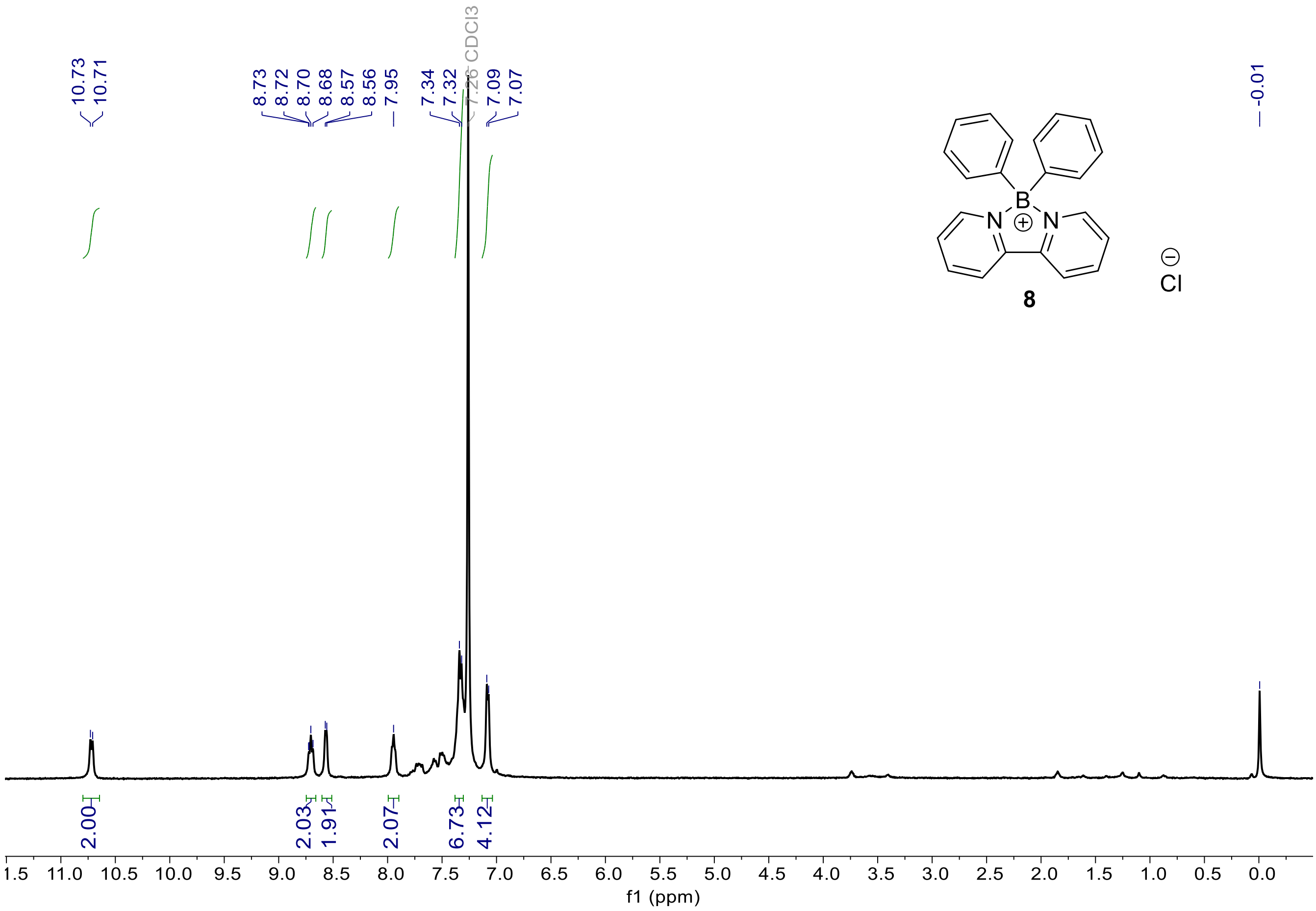

Supplementary Figure 13. ^{1}H NMR of **8** in $CDCl_3$

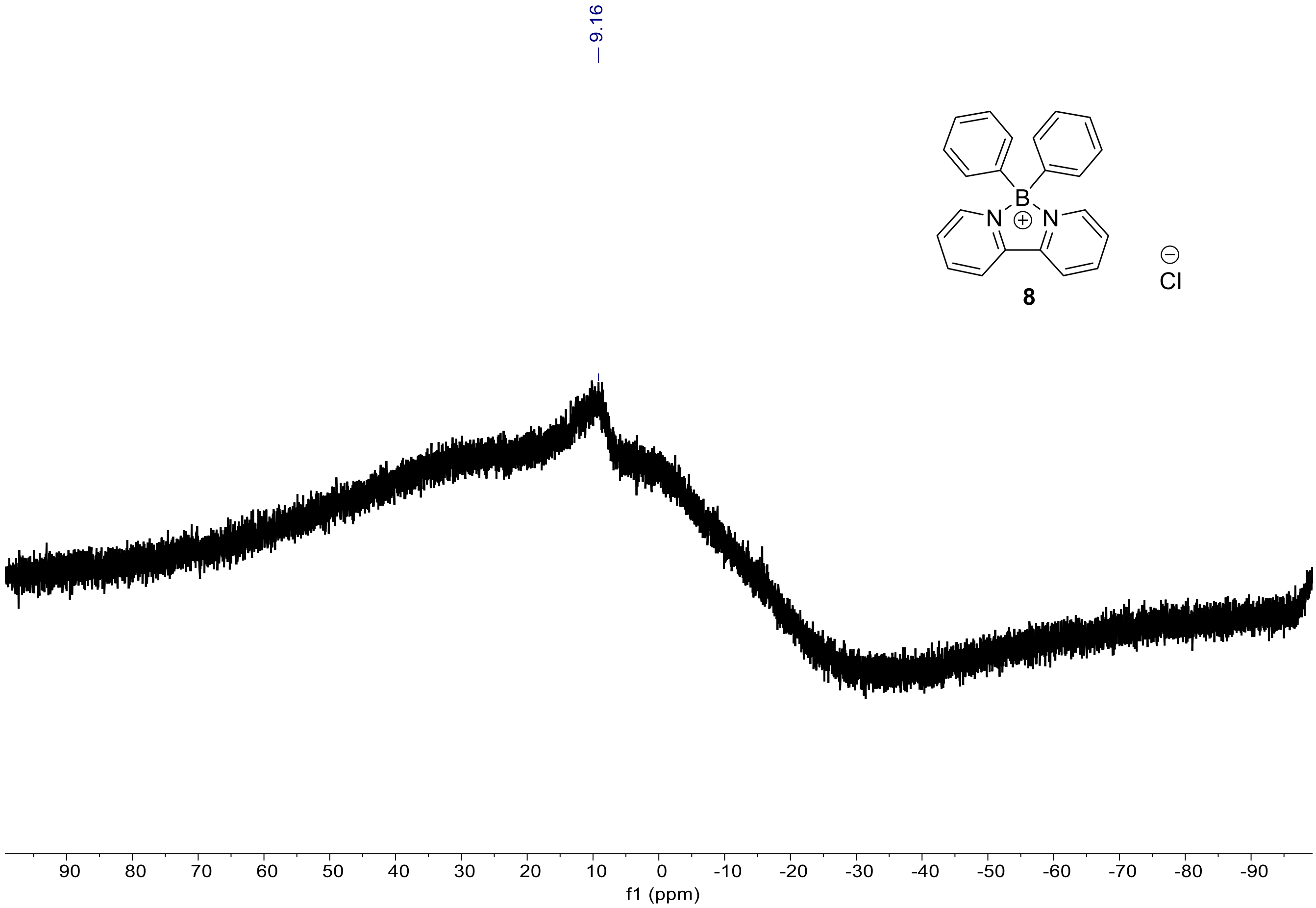

Supplementary Figure 14. ^{11}B NMR of **8** in $CDCl_3$

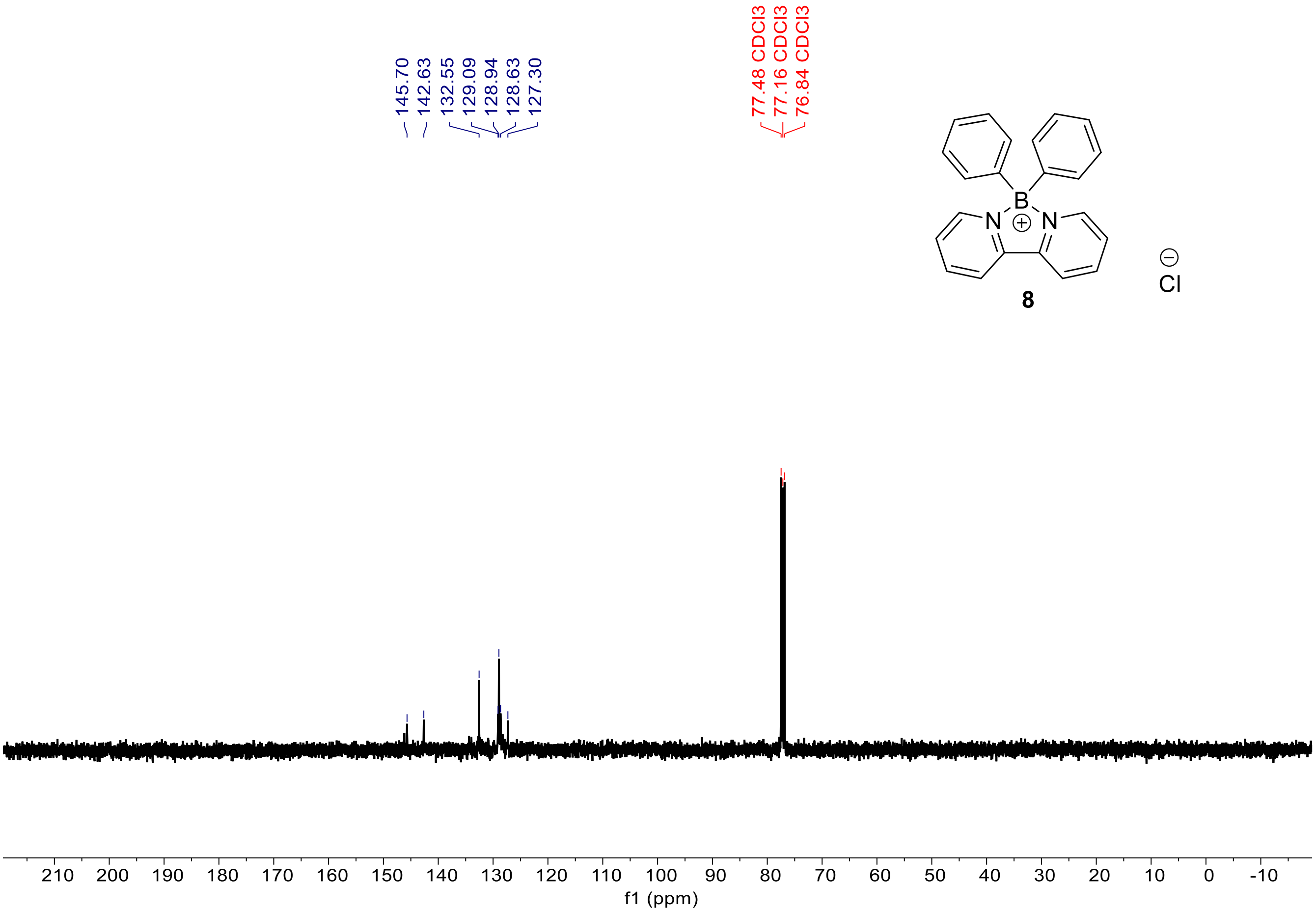

Supplementary Figure 15. $^{13}C\{^{1}H\}$ NMR of **8** in $CDCl_3$

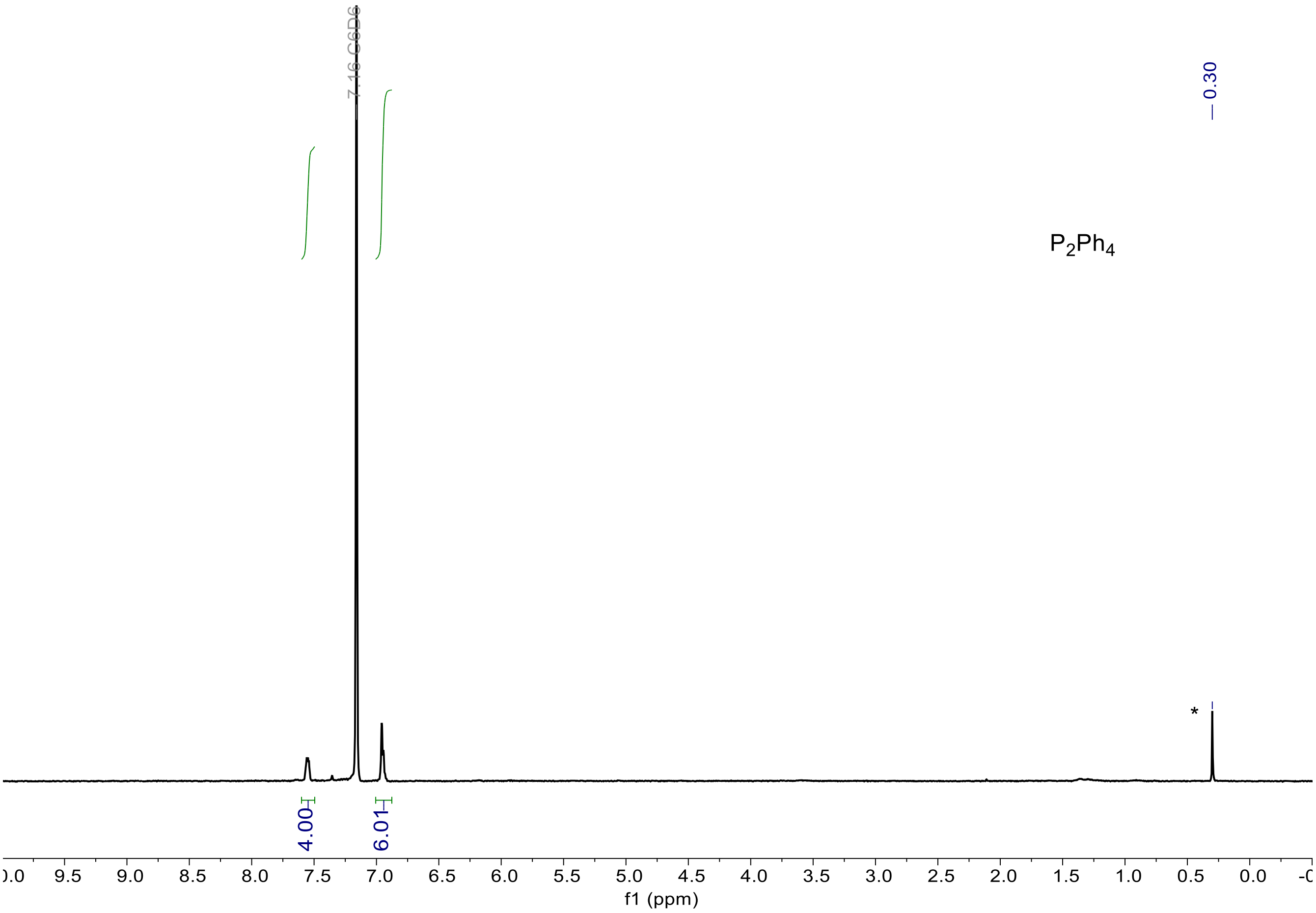

Supplementary Figure 16. ^{1}H NMR of **P_2Ph_4** in C_6D_6 (*: silicone grease from SPS purified n-hexane)

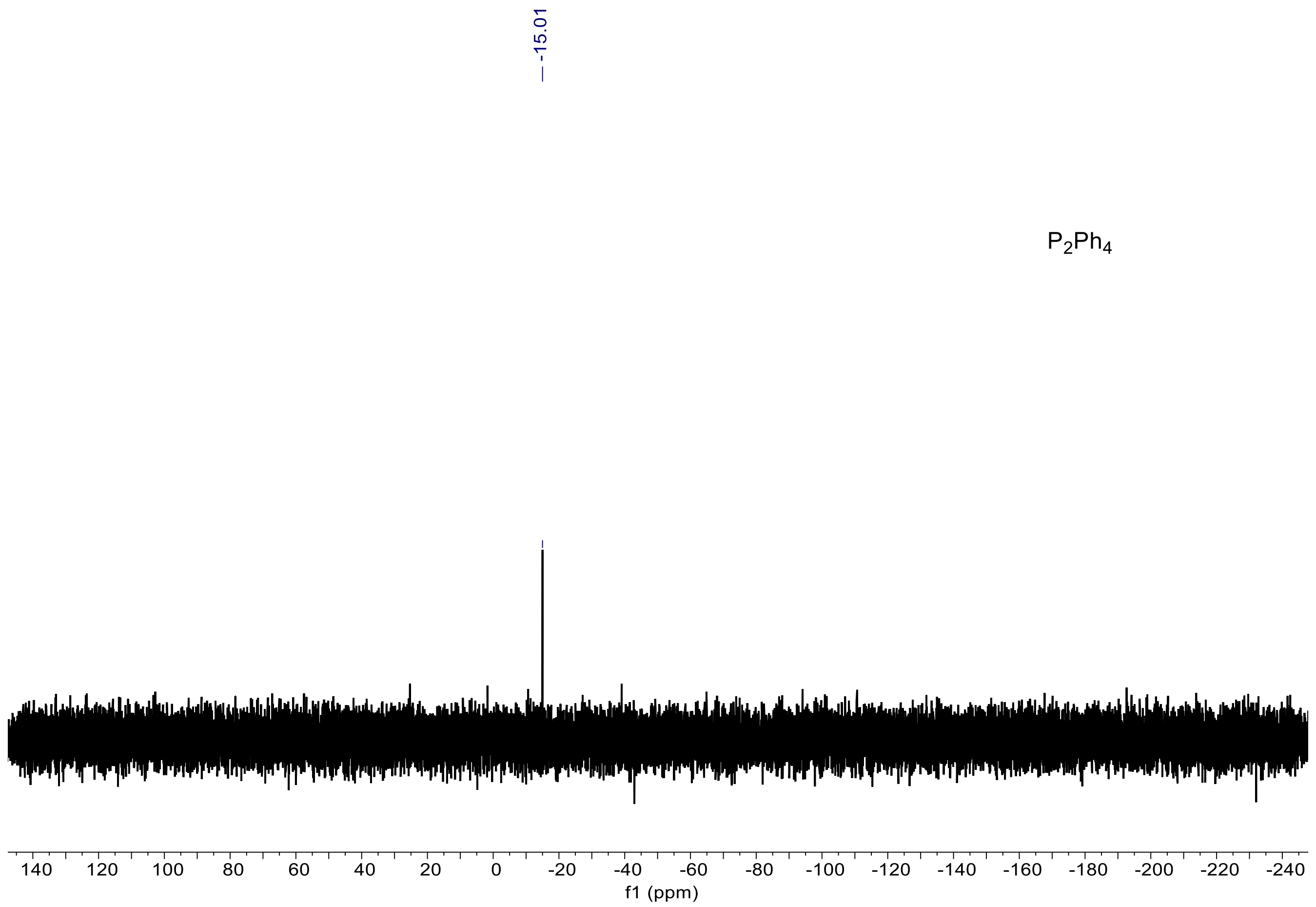

Supplementary Figure 17. $^{31}P\{^1H\}$ NMR of $\mathbf{P_2Ph_4}$ in C_6D_6

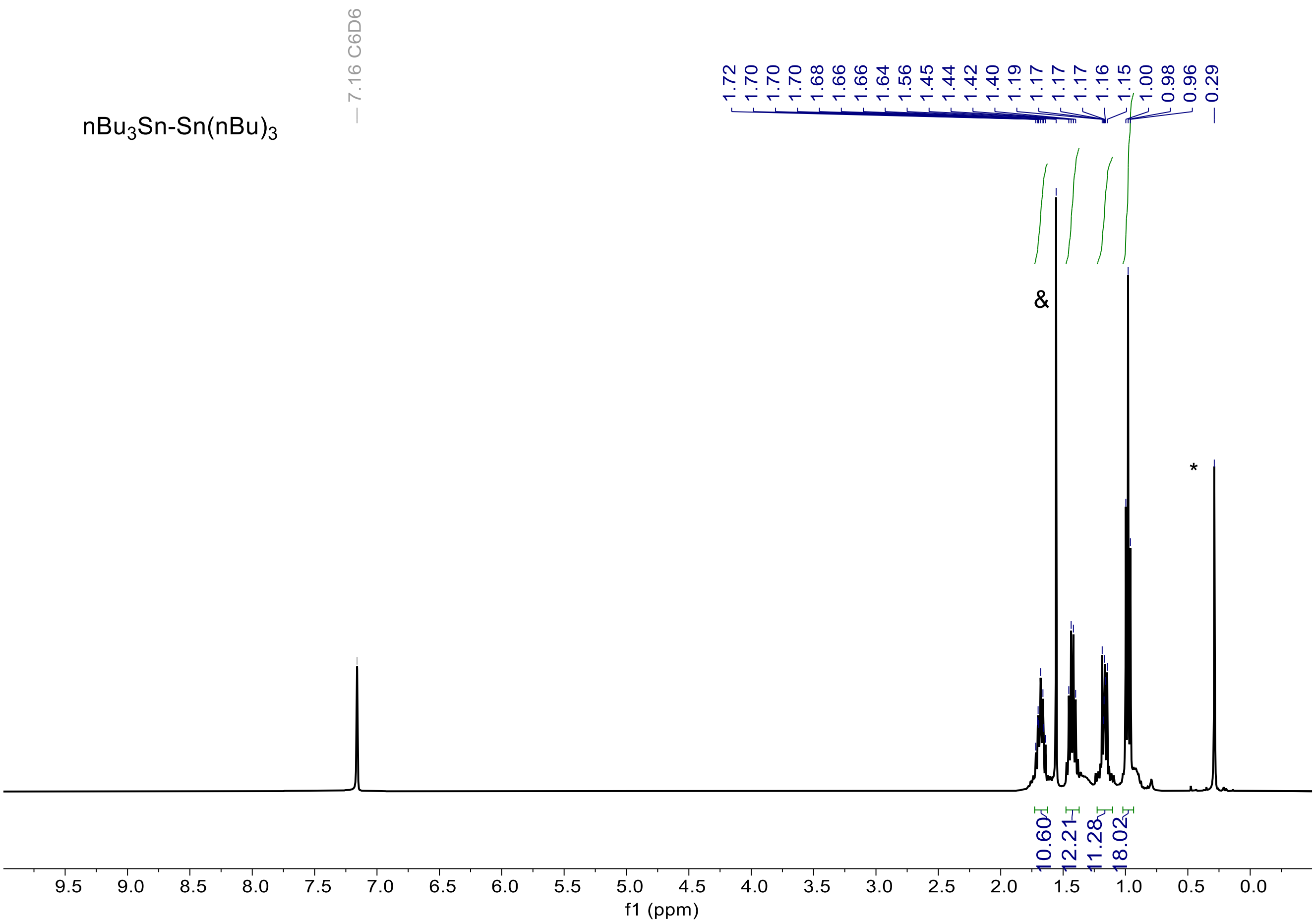

Supplementary Figure 18. ^1H NMR of **nBu$_6$Sn$_2$** in C_6D_6 (*: silicone grease from SPS purified n-hexane; &: acetone from deuterated solvent)

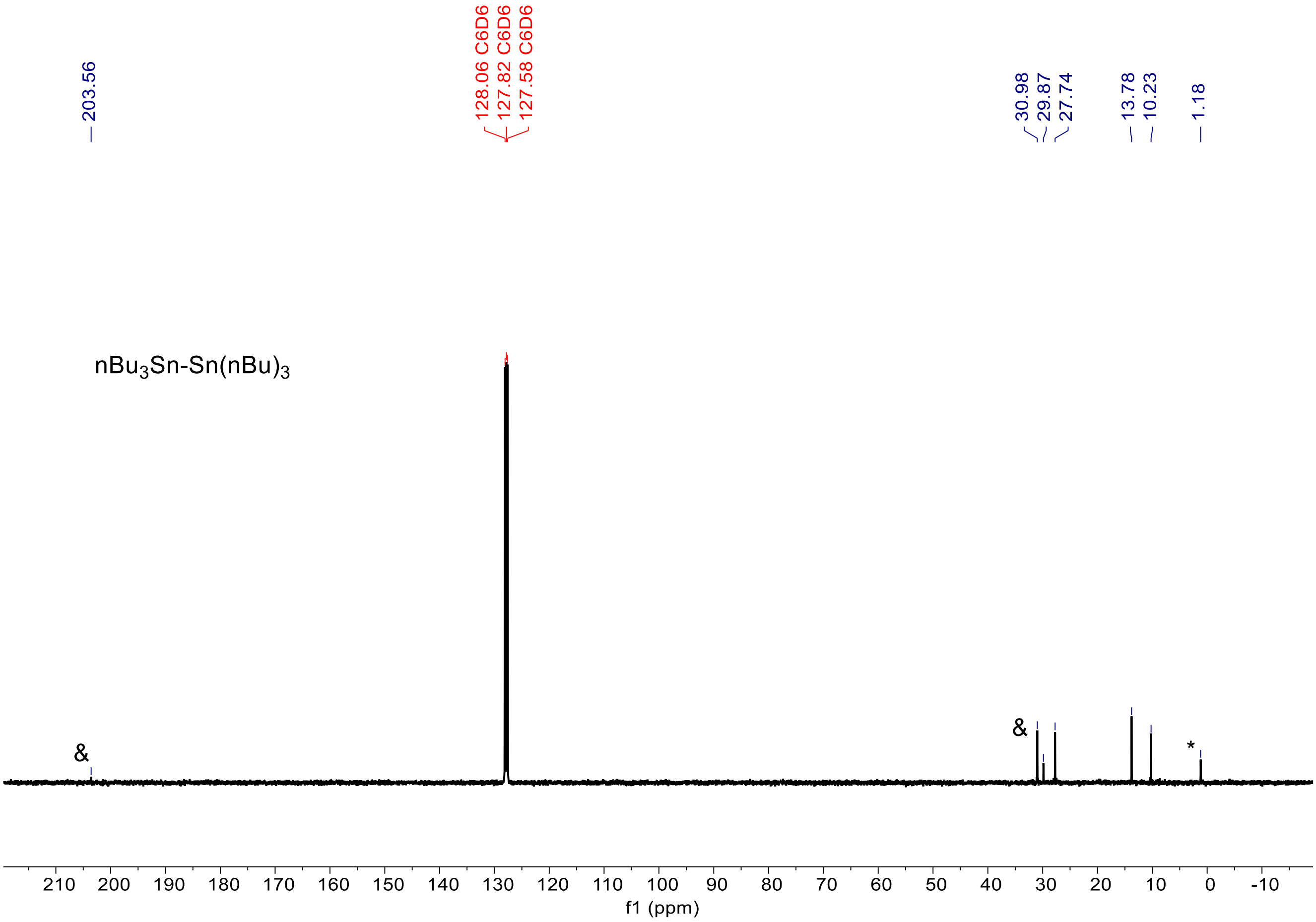

Supplementary Figure 19. $^{13}C\{^{1}H\}$ NMR of **nBu_6Sn_2** in C_6D_6 (*: silicone grease from SPS purified n-hexane; &: acetone from deuterated solvent)

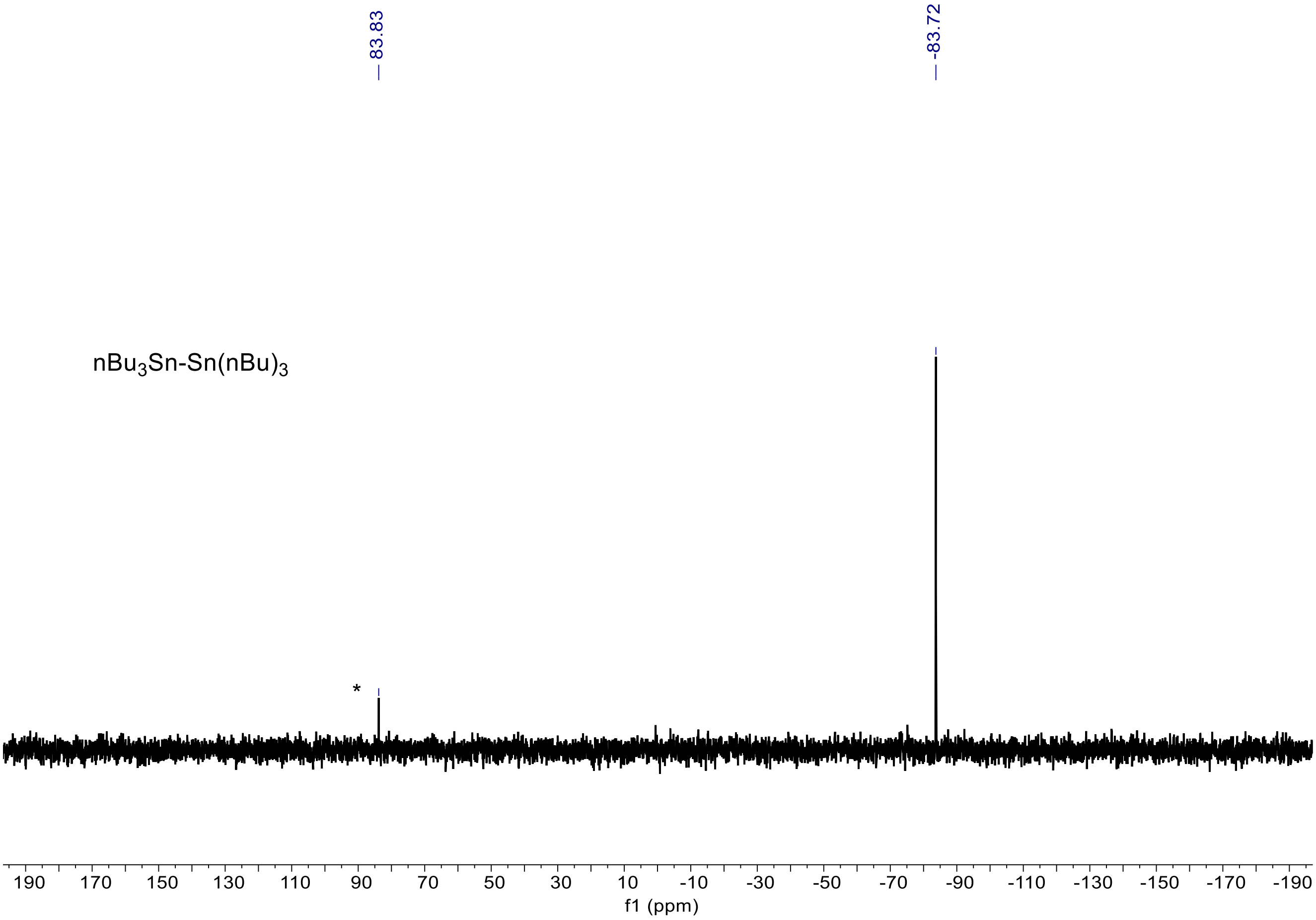

Supplementary Figure 20. $^{119}Sn\{^{1}H\}$ NMR of **nBu_6Sn_2** in C_6D_6 (*: unknow impurity)

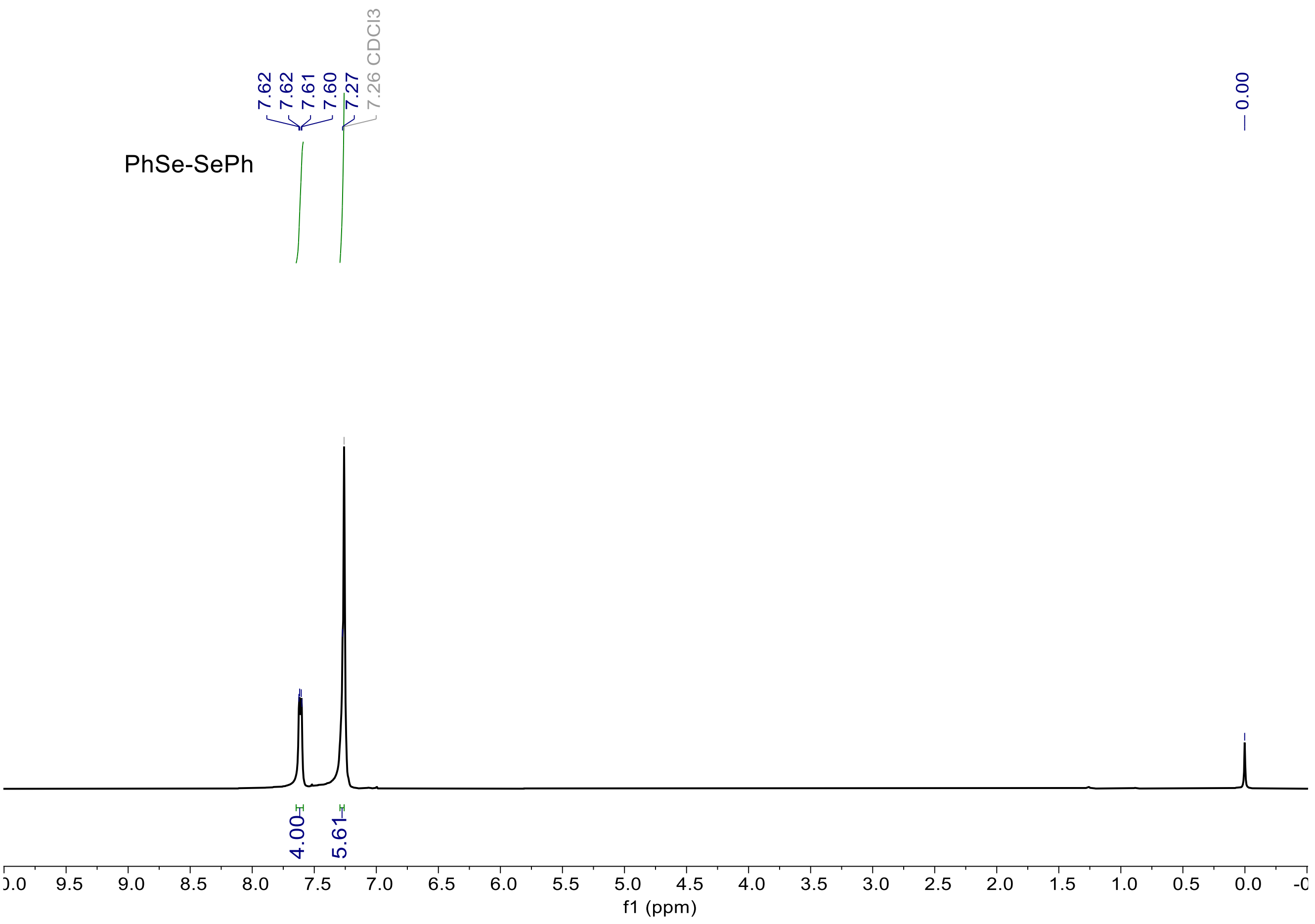

Supplementary Figure 21. ^{1}H NMR of **Ph_2Se_2** in $CDCl_3$

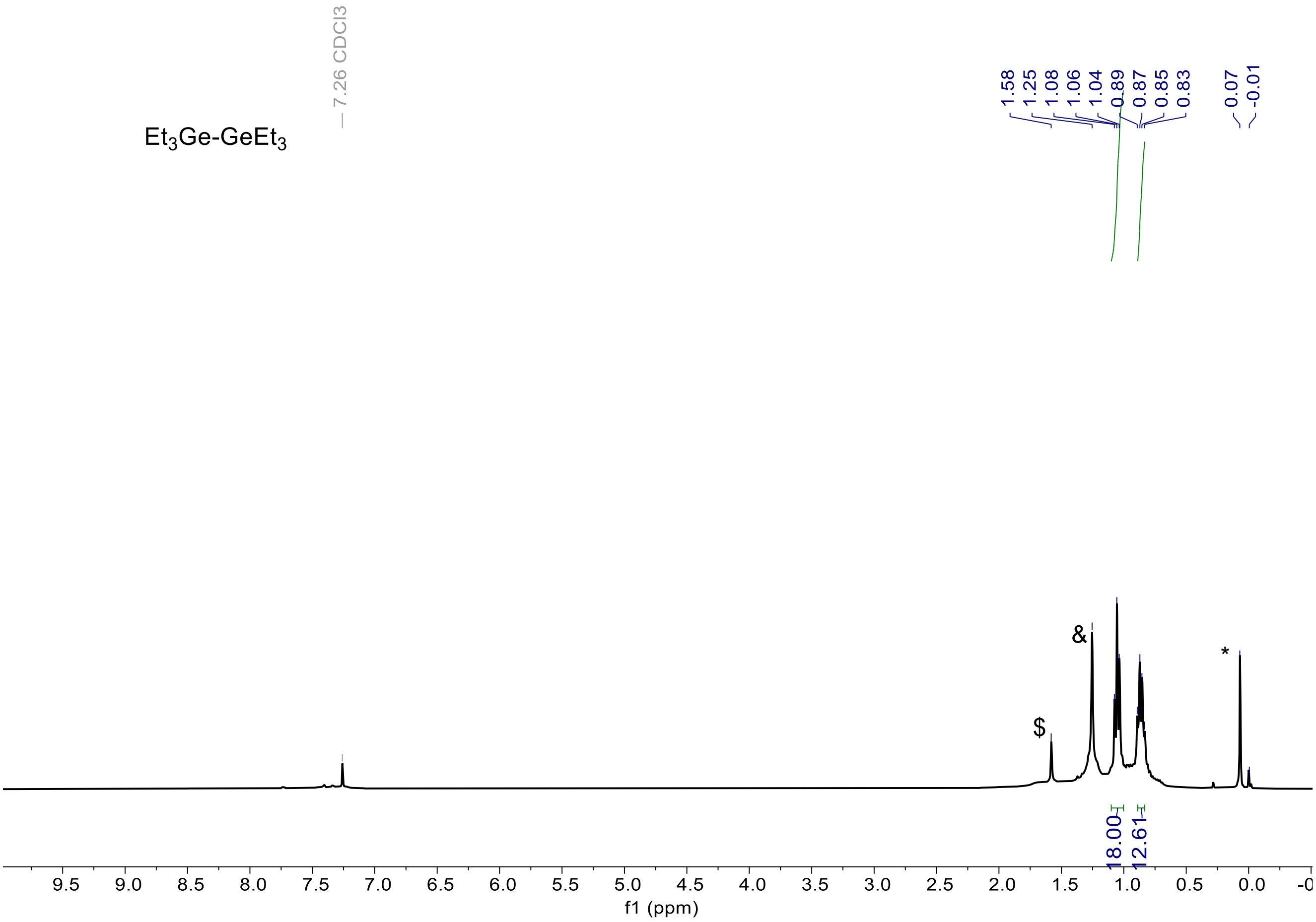

Supplementary Figure 22. ^{1}H NMR of **Et_6Ge_2** in $CDCl_3$ (*: silicone grease from SPS purified n-hexane; &: H grease; $: water from deuterated solvent)

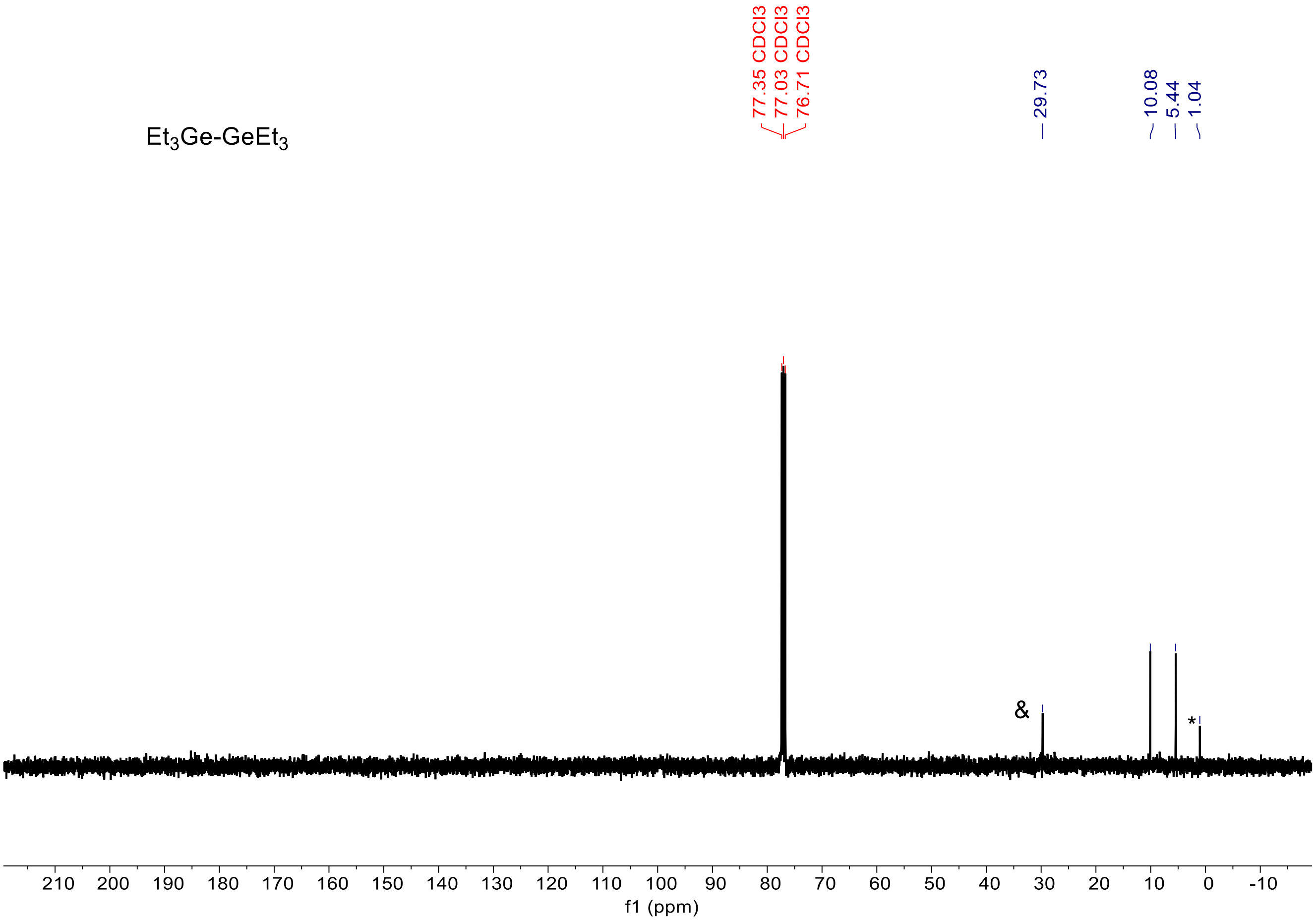

Supplementary Figure 23. $^{13}C\{^{1}H\}$ NMR of **Et_6Ge_2** in $CDCl_3$(*: silicone grease from SPS purified n-hexane; &: H grease)

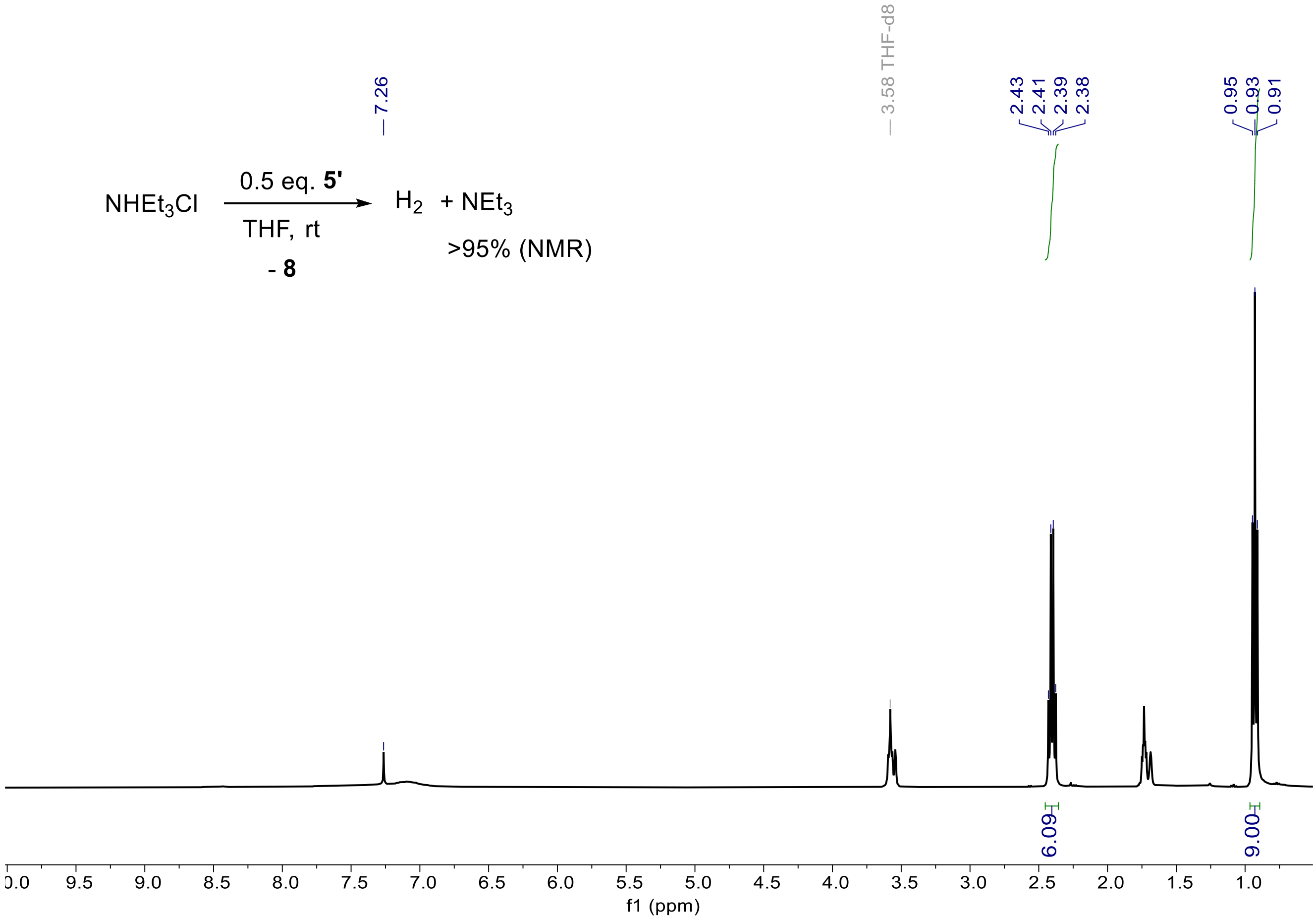

Supplementary Figure 24. ^{1}H NMR of **$NHEt_3Cl$ reactions** in THF-d_8

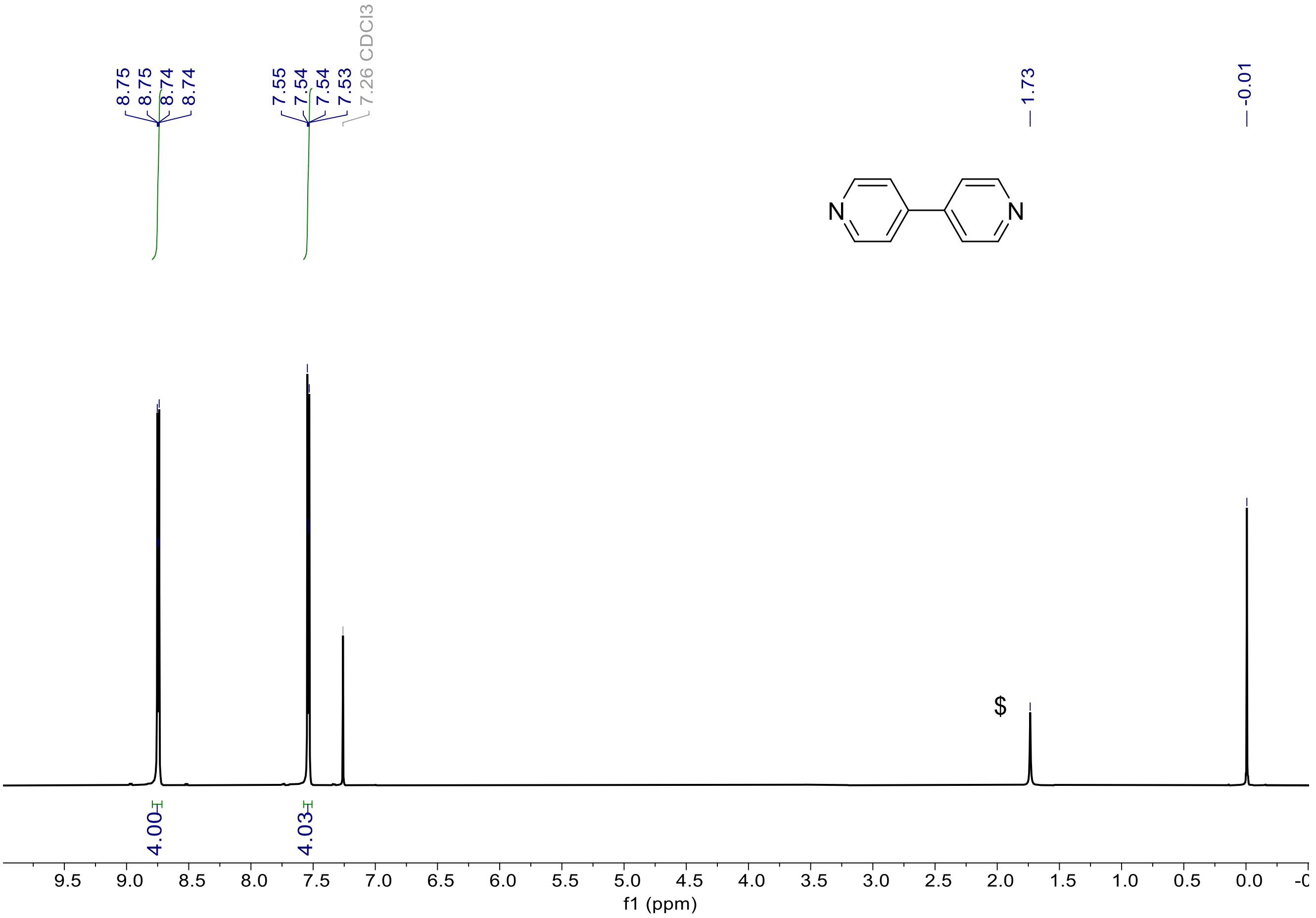

Supplementary Figure 25. ^{1}H NMR of **4,4'-bipyridine** in $CDCl_3$ ($: water from deuterated solvent)

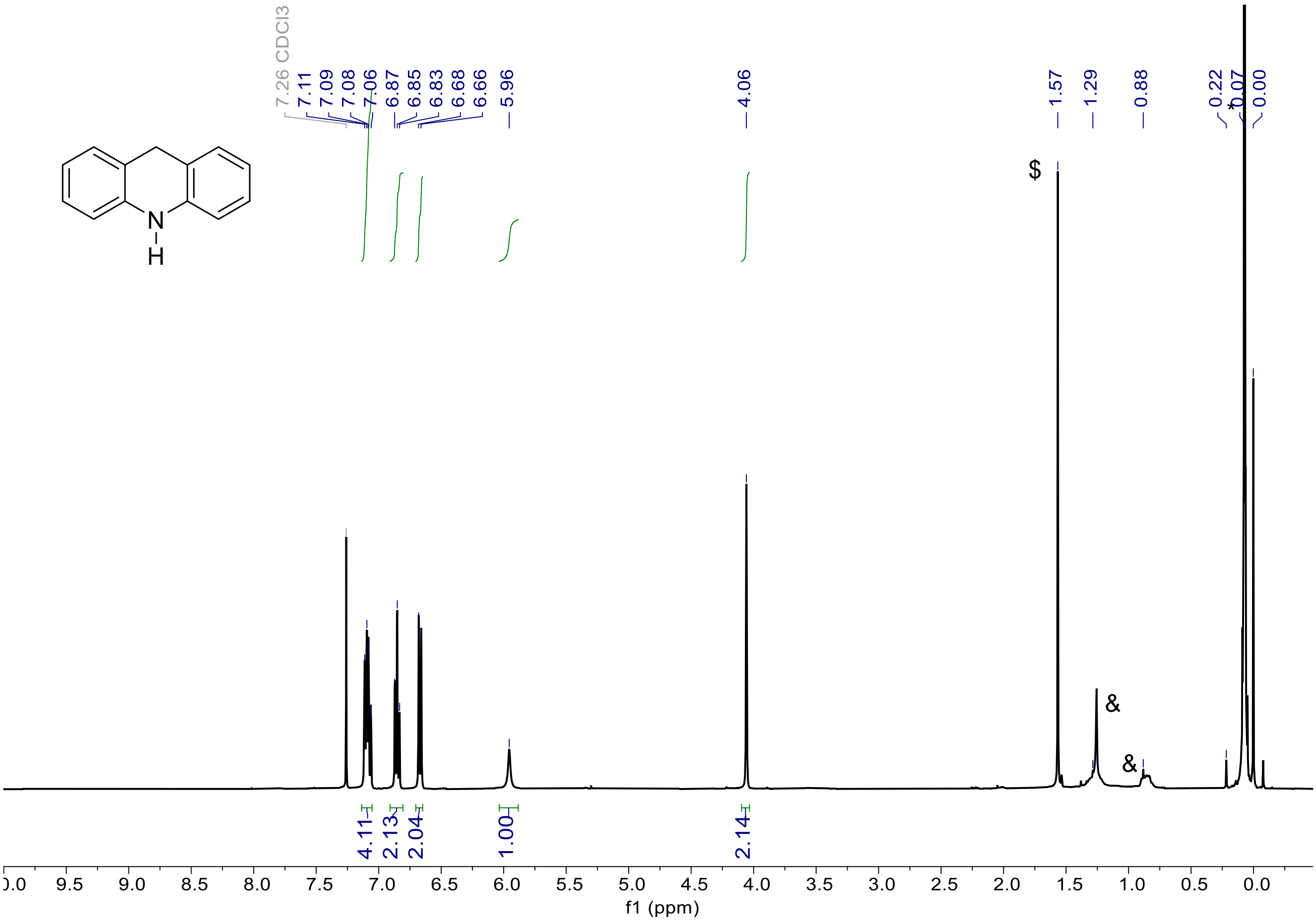

Supplementary Figure 26. ^{1}H NMR of **Acridan** in $CDCl_3$ (*: silicone grease from SPS purified n-hexane; &: H grease; $: water from deuterated solvent)

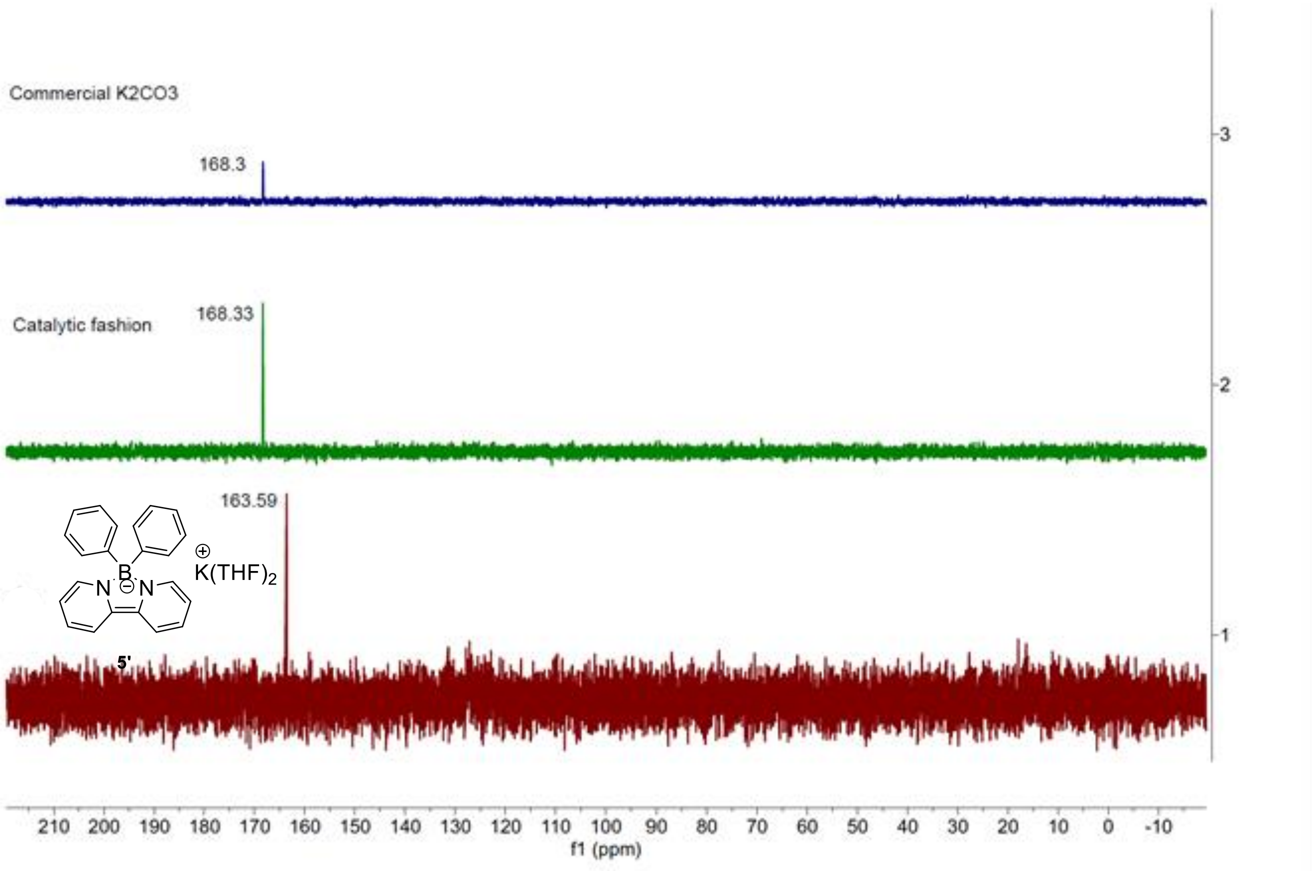

Supplementary Figure 27. $^{13}C\{^{1}H\}$ NMR of **CO_2 reactions** in D_2O

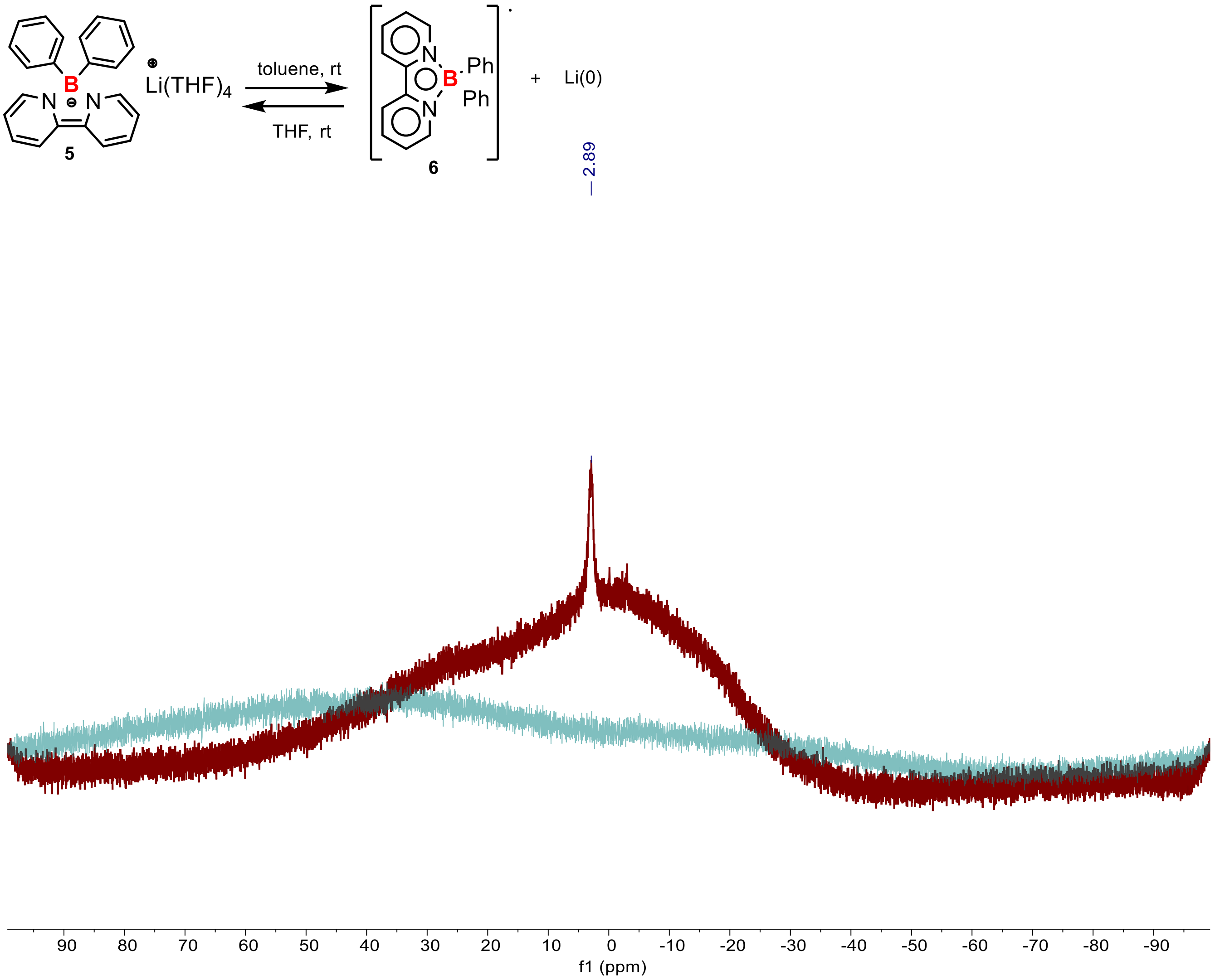

Supplementary Figure 28. ^{11}B NMR of **5** in toluene/THF. Blue: toluene (EPR active); Red: removal of toluene and add THF.

Gas Chromatography

Gas products were analyzed by online gas chromatography (GC2060).

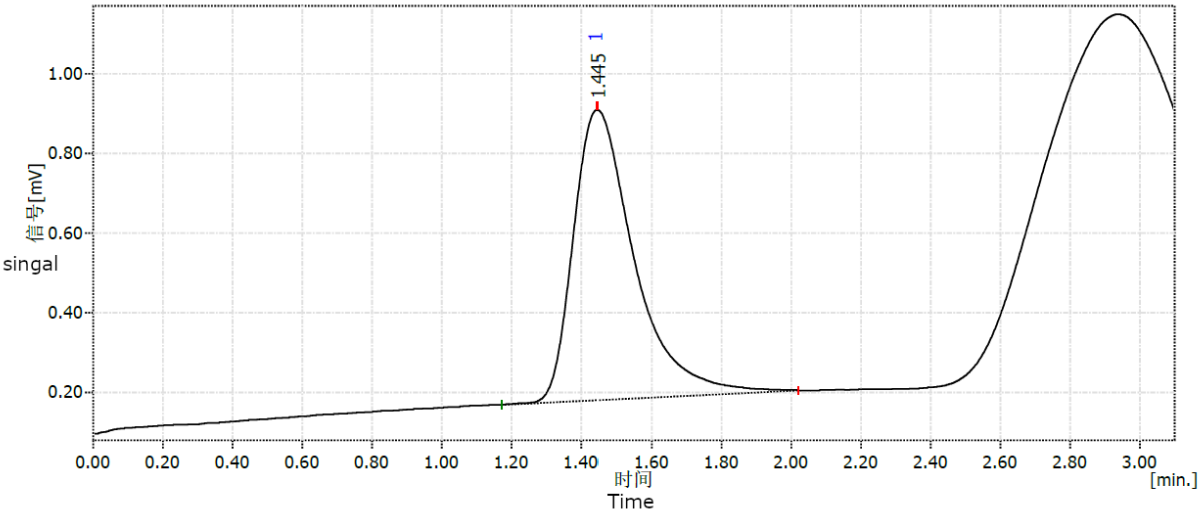

No. 序号	Name 组份名称	Retention time 保留时间(min)	peak area 峰面积	concentration 浓度()	area percentage 面积百分比	concentration percentage 浓度百分比
1		1.4450	8.7413		100.0000	
sum 合计			8.7413	0.0000	100	0.0000

Supplementary Figure 29. GC spectra of **$NHEt_3Cl$ reaction**.

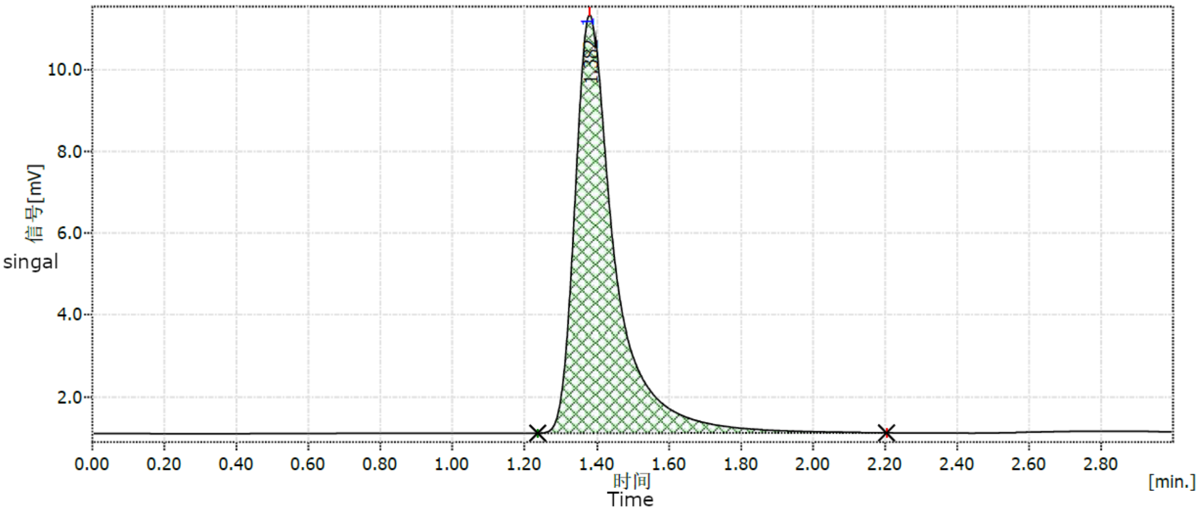

No.<br>序号	Name<br>组份名称	Retention time<br>保留时间(min)	peak area<br>峰面积	concentration<br>浓度()	area percentage<br>面积百分比	concentration percentage<br>浓度百分比
1		1. 3817	77. 9071		100. 0000	
sum<br>合计			77. 9071	0. 0000	100	0. 0000

Supplementary Figure 30. GC spectra of pure $\mathbf{H_2}$.

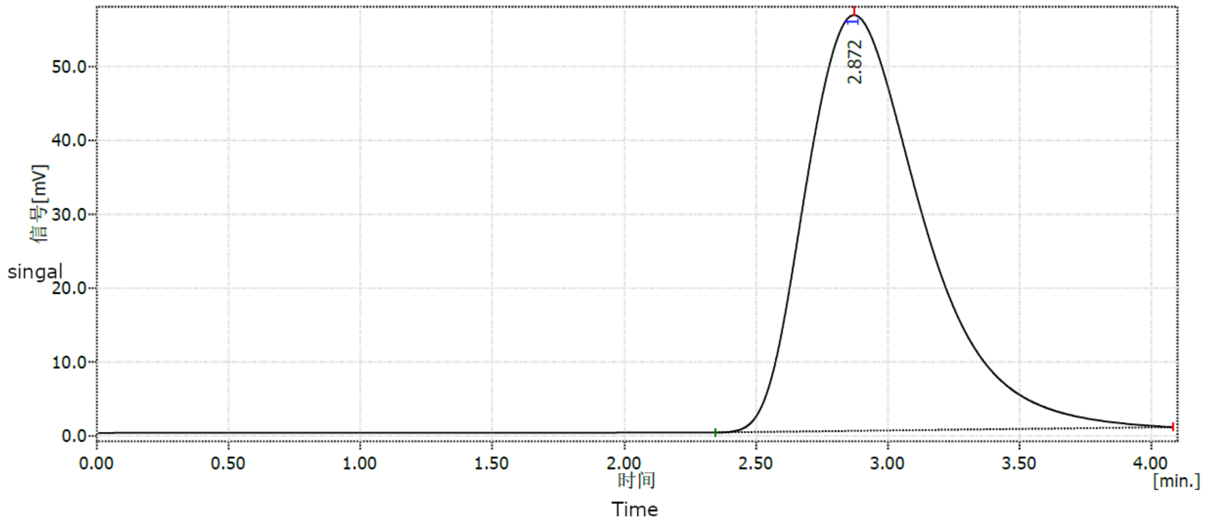

No. 序号	Name 组份名称	Retention time 保留时间(min)	peak area 峰面积	concentration 浓度()	area percentage 面积百分比	concentration percentage 浓度百分比
1		2.8717	1751.7290	0.0000	0.0000	
sum 合计			1751.7290	0.0000	100	0.0000

Supplementary Figure 31. GC spectra of pure **Air of the lab**.

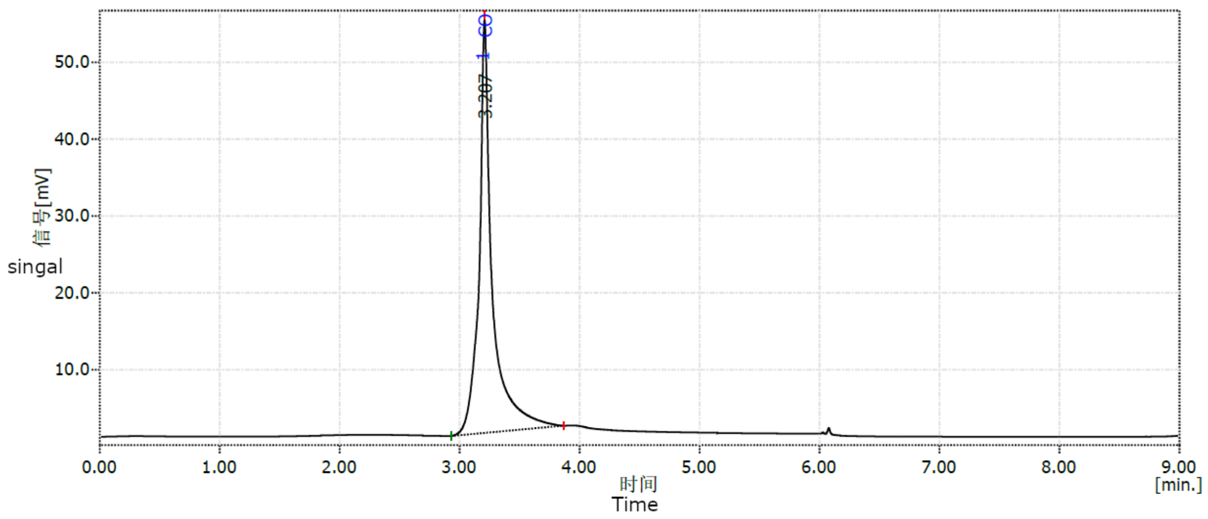

No. 序号	Name 组份名称	Retention time 保留时间(min)	peak area 峰面积	concentration 浓度()	area percentage 面积百分比	concentration percentage 浓度百分比
1	CO	3. 2067	411. 8424	0. 1663	100. 0000	100. 0000
sum 合计			411. 8424	0. 1663	100	1. 0000

Supplementary Figure 32. GC spectra of **CO_2 reactions(5')**.

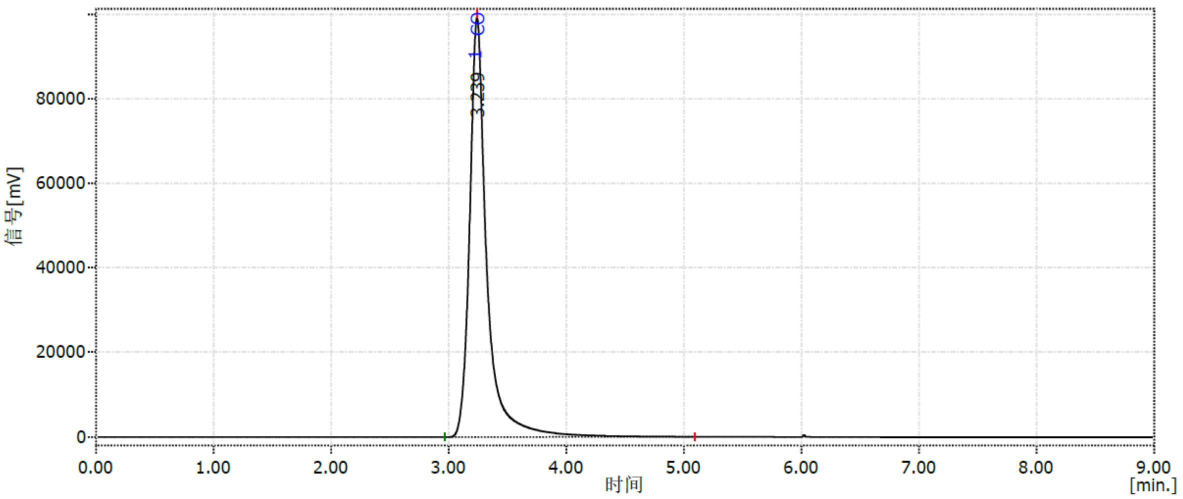

序号	组份名称	保留时间(min)	峰面积	浓度()	面积百分比	浓度百分比
1	CO	3.2392	1008521.0000	407.3193	100.0000	100.0000
合计			1008521.0000	407.3193	100	1.0000

Supplementary Figure 33. GC spectra of pure **CO**.

Cyclic Voltammetry

Cyclic voltammetry (CV) was tested using CH instruments CHI660 electrochemical workstation. All experiments were carried out under an atmosphere of argan in the three-electrode cell (glassy carbon used as working electrode, platinum wire electrode used as counter electrode, and silver electrode used as reference electrode) with anhydrous THF solution containing 0.1 M [nBu_4N][PF_6] (dried over 3 Å molecular sieves for one week before test). Ferrocene/ferrocenium couple was used as an internal standard.

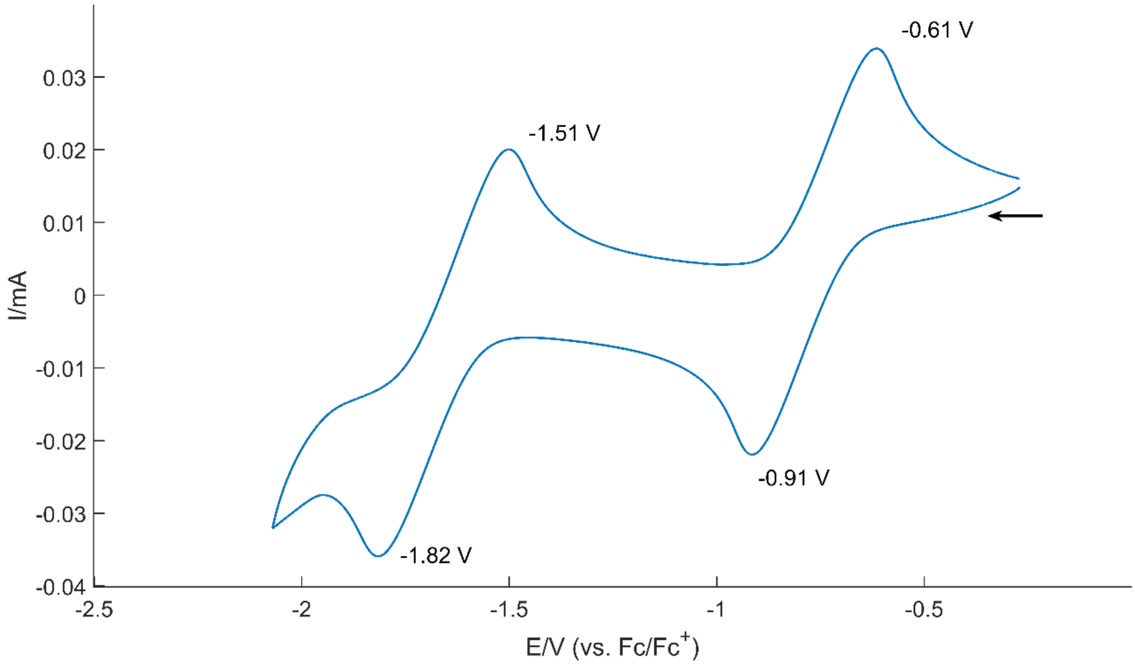

Supplementary Figure 34 Cyclic voltammograms (CVs) of **6** in THF solution containing 0.1 M [nBu4N][PF6]) at room temperature (scan rate: 100 mV/s).

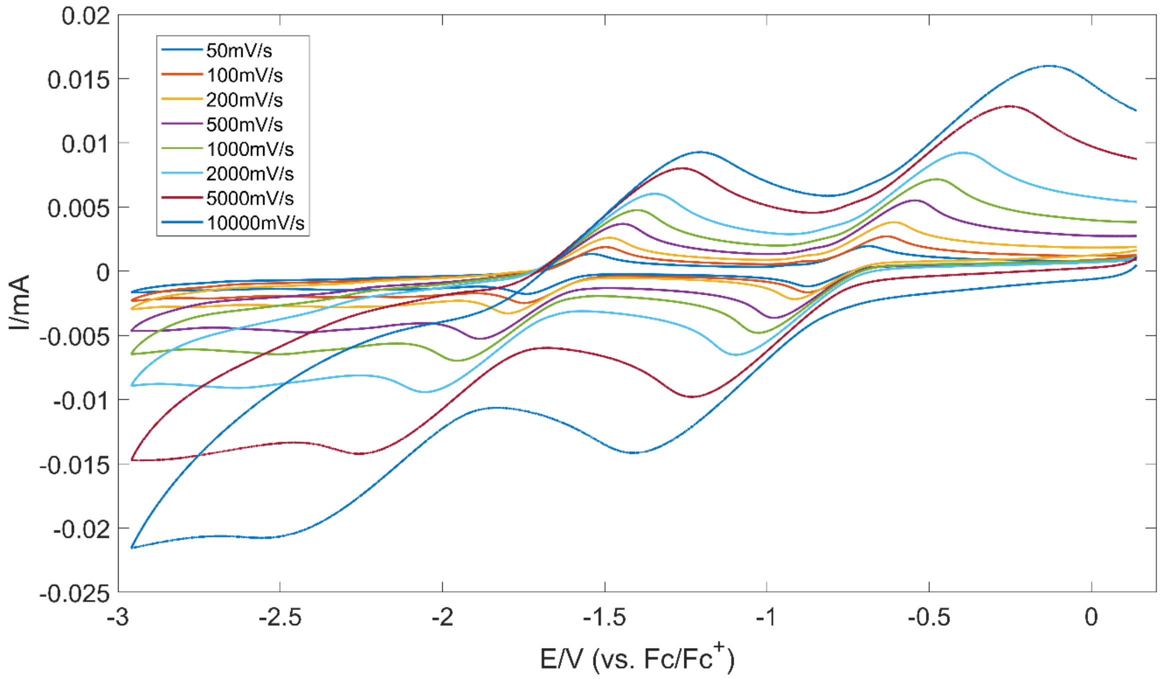

Supplementary Figure 35 Cyclic voltammograms (CVs) of **6** in THF solution containing 0.1 M [nBu4N][PF6]) at room temperature with different scan rates.

EPR Spectroscopy

EPR spectra were acquired on ADANI SPINSCAN X EPR Spectrometer. All samples were prepared and tested under argon conditions. Easyspin (5.2.35) [7] was used to simulate the spectra. Microwave frequency was 9.450400 GHz, and power was 14.581mW.

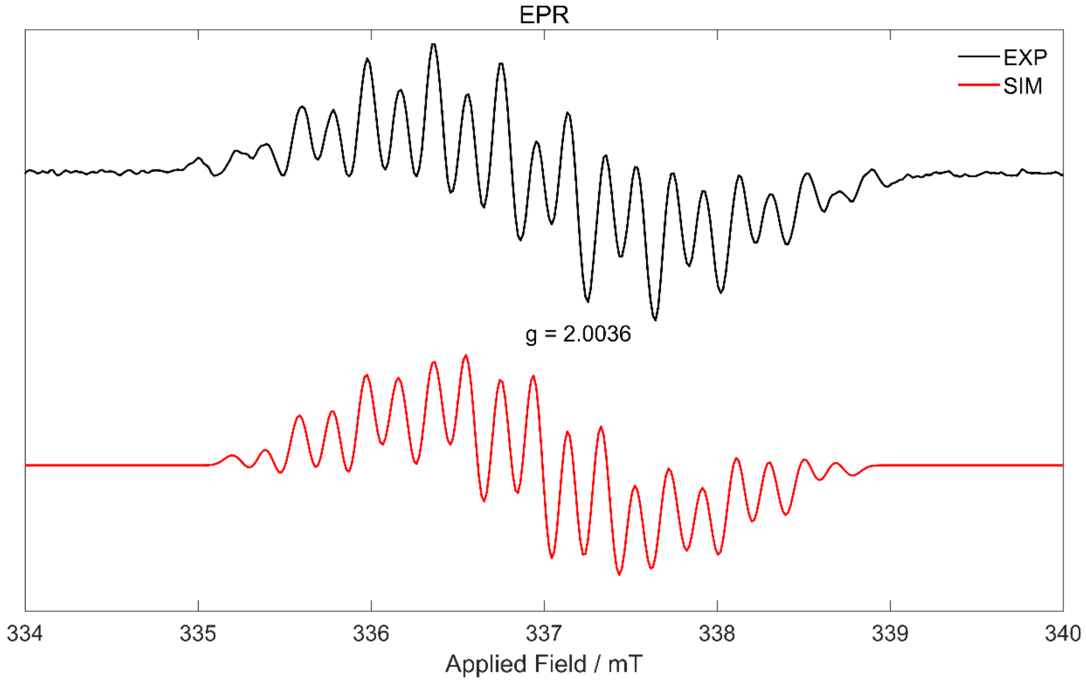

Supplementary Figure 36. EPR spectra of compound **6** in toluene solution at room temperature, (^{11}B)/(^{10}B) = 4/1, a(^{10}B) = 0.05 G, a(^{11}B) = 0.16 G, a(^{14}N) = 3.57 G, a(^{1}H) = 1.01, 7.77, 8.89, 10.77 G).

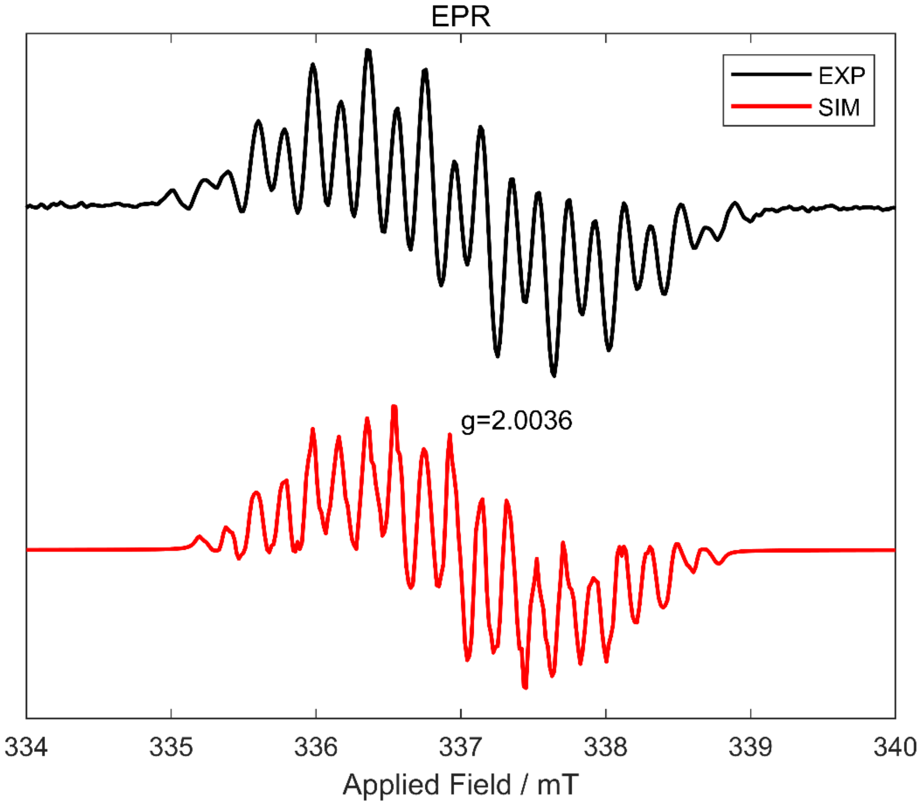

Supplementary Figure 37. EPR spectra of compound **6** in toluene solution at room temperature, a(^{11}B) = 0.22 G, a(^{14}N) = 3.58 G, a(^{1}H) = 1.01, 7.77, 8.89, 10.77 G).

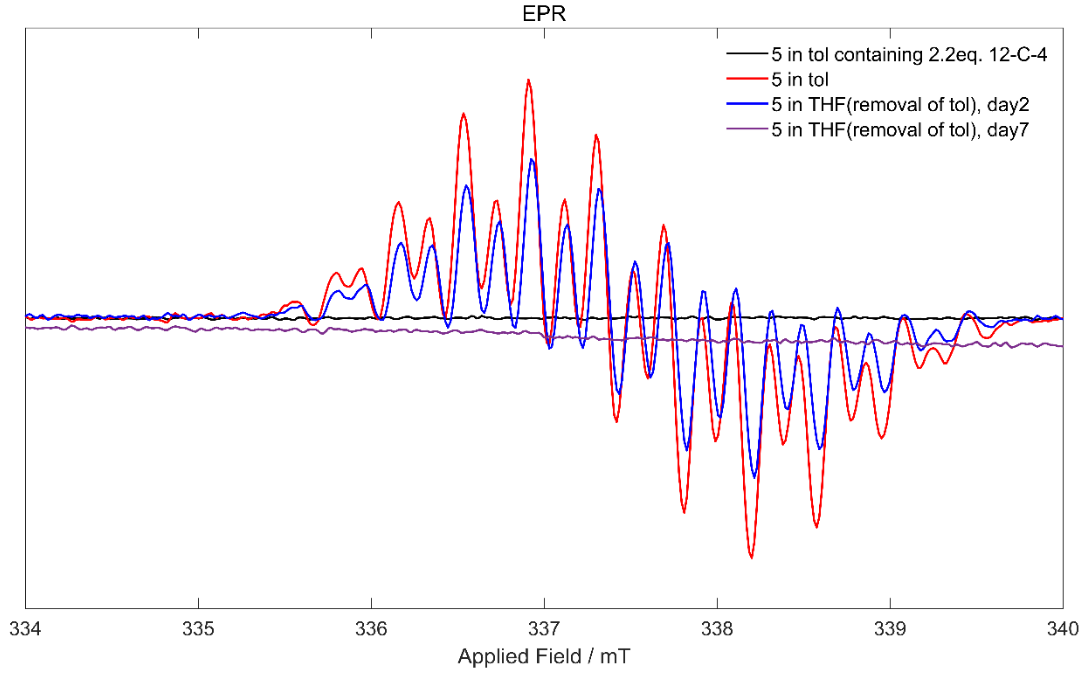

Supplementary Figure 38. EPR spectra of control experiments.

Powder XRD Analysis

Powder XRD Analysis was acquired on Bruker D2 Phaser. The sample was prepared as follows: during the synthesis of [(bipy)BPh_2] (**6**), the insoluble black solids were collected and washed with THF (6 mL) and dried under a fine vacuum as grey powder. Samples were stored in argon and quickly tested in air. High-intensity peaks appeared at 2θ = 32.50°,36.20° were in good agreement with the reported data of lithium powder.[8]

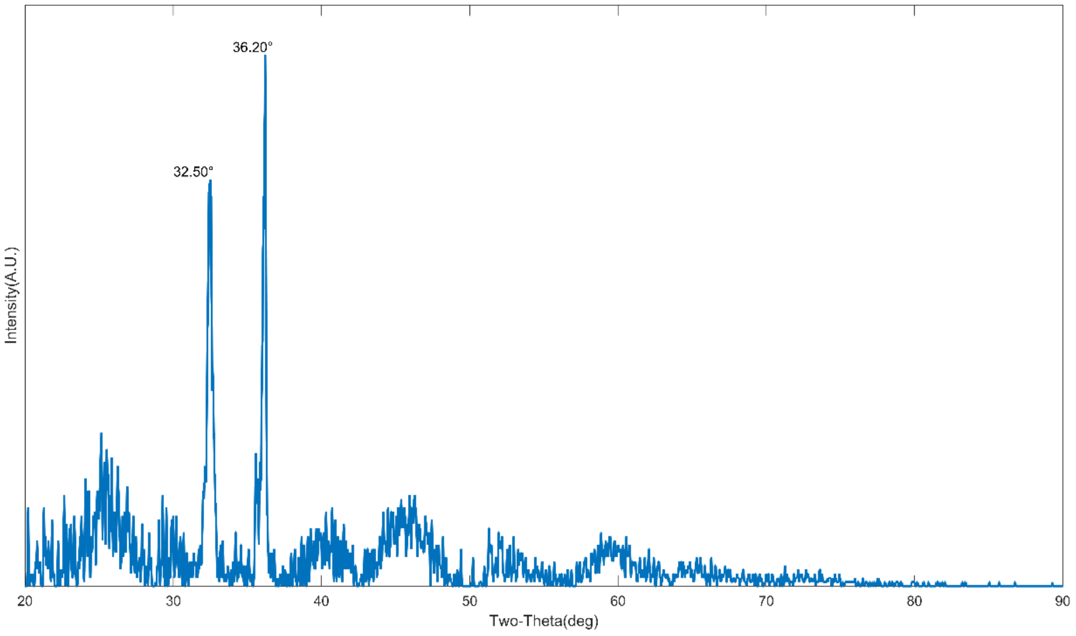

Supplementary Figure 39. Powder XRD of the residue formed in the synthesis of **6** confirmed as Li(0).

X-ray photoelectron spectroscopy (XPS)

XPS Analysis was acquired on Thermo Scientific K-Alpha.

Sample Exp. was prepared as follows: during the synthesis of [(bipy)BPh_2] (**6**), the insoluble black solids were collected and washed with THF (6 mL) and dried under a fine vacuum as grey powder.

Sample Cml. was from Sigma-Aldrich 499811-100G, Lithium granular, 99% trace metals basis.

Sample Exp. + Cml. was a mixture of Sample Exp. and Sample Cml.

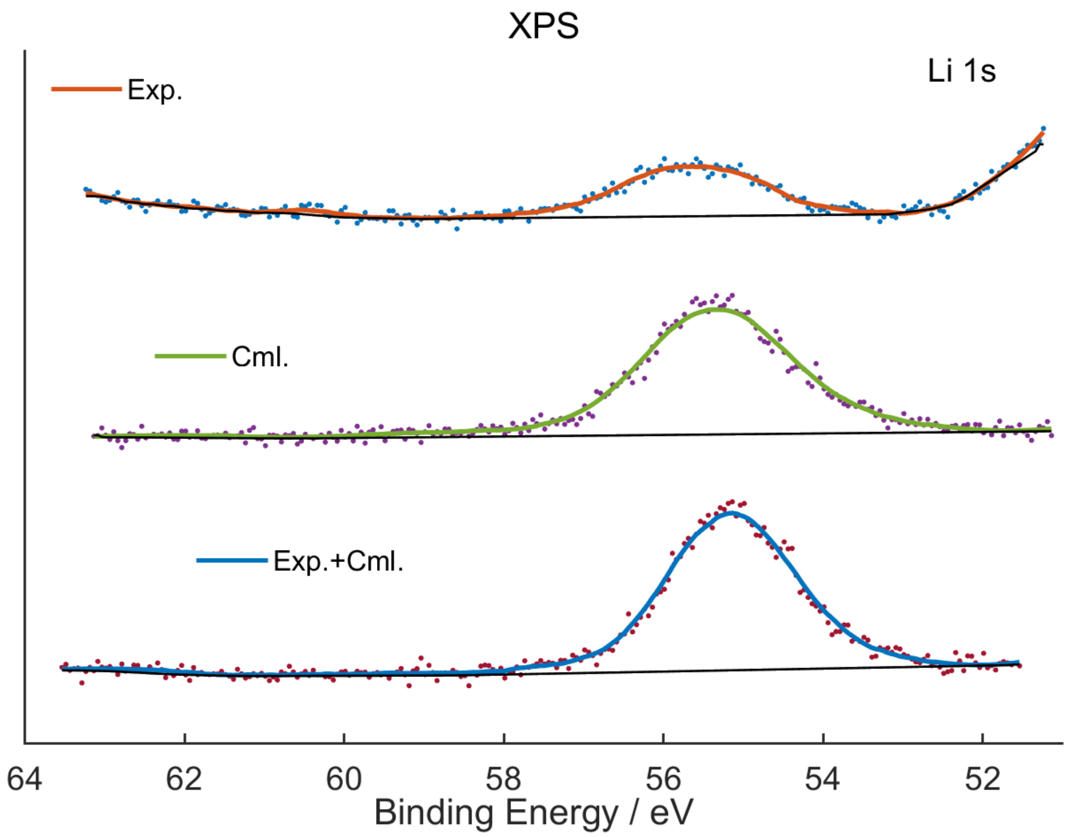

Supplementary Figure 40. XPS results. Binding Energy was referred to C1s = 284.8 eV.

Sample Exp.:

Name	Start BE	Peak BE	End BE	Height CPS	FWHM eV	Area (P) CPS.eV	Area (N)	Atomic %	Peak Type	Q	SF	TXFN	Background
C1s	298.03	283.64	279.13	23161.95	2.24	62374.5	873.99	73.13	Standard	1	1	1	Smart
Li1s	62.03	54.42	50.13	641.26	2.11	1599.41	321.05	26.87	Standard	1	0.061	1	Smart

Sample Cml.:

Name	Start BE	Peak BE	End BE	Height CPS	FWHM eV	Area (P) CPS.eV	Area (N)	Atomic %	Peak Type	Q	SF	TXFN	Background
C1s	298.03	283.74	279.13	32835.63	1.31	63488.48	889.65	51.45	Standard	1	1	1	Smart
Li1s	62.03	54.32	50.13	1723.78	1.74	4183.27	839.66	48.55	Standard	1	0.061	1	Smart

Sample Exp. + Cml.:

Name	Start BE	Peak BE	End BE	Height CPS	FWHM eV	Area (P) CPS.eV	Area (N)	Atomic %	Peak Type	Q	SF	TXFN	Background
C1s	298.03	283.34	279.13	28731.32	1.3	59023.32	826.87	48	Standard	1	1	1	Smart
Li1s	62.03	53.73	50.13	2091.28	1.73	4464.37	895.79	52	Standard	1	0.061	1	Smart

ICP-OES Study

Inductively Coupled Plasma Optical Emission spectroscopy (ICP-OES) was recorded using The OPtima 8000 spectrometer.

1. Detection of Li^+

The sample was collected from the following reaction. The insoluble black solids were collected, washed with THF (6 mL), and dried under a fine vacuum as grey powder. Compound **5** (1.5 mg) was quenched with deionized water (1 mL).

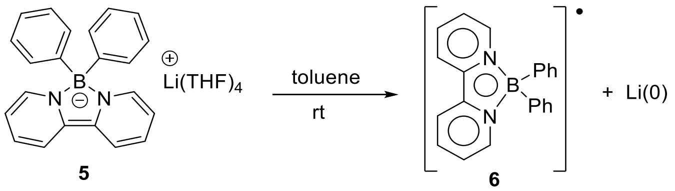

5 **6**

Replicate Data: DL

Repl#	Analyte	Net Intensity	Corrected Intensity	Calib. Conc. Units	Sample Conc. Units	Analysis Time
1	Li 670.784	8537604.9	8533058.4	0.4205 mg/L	0.4205 mg/L	4:35:48 PM
2	Li 670.784	8512449.5	8507903.0	0.4193 mg/L	0.4193 mg/L	4:35:53 PM
3	Li 670.784	8399849.0	8395302.5	0.4137 mg/L	0.4137 mg/L	4:35:55 PM
4	Li 670.784	8357615.5	8353069.0	0.4116 mg/L	0.4116 mg/L	4:35:57 PM
5	Li 670.784	8633311.1	8628764.6	0.4252 mg/L	0.4252 mg/L	4:36:00 PM
6	Li 670.784	8680005.7	8675459.2	0.4275 mg/L	0.4275 mg/L	4:36:02 PM
7	Li 670.784	8377892.9	8373346.4	0.4126 mg/L	0.4126 mg/L	4:36:05 PM
8	Li 670.784	8659146.1	8654599.6	0.4265 mg/L	0.4265 mg/L	4:36:07 PM
9	Li 670.784	8538119.2	8533572.7	0.4205 mg/L	0.4205 mg/L	4:36:10 PM
10	Li 670.784	8307158.2	8302611.6	0.4091 mg/L	0.4091 mg/L	4:36:12 PM

Mean Data: DL

Analyte	Mean Corrected Intensity	Calib. Conc. Units	Std.Dev.	Sample Conc. Units	Std.Dev.	RSD
Li 670.784	8495768.7	0.4187 mg/L	0.00659	0.4187 mg/L	0.00659	1.57%

Replicate Data: DI water

Repl#	Analyte	Net Intensity	Corrected Intensity	Calib. Conc. Units	Sample Conc. Units	Analysis Time
1	Li 670.784	53758.9	49212.4	0.0024 mg/L	0.0024 mg/L	3:57:51 PM
2	Li 670.784	51944.1	47397.5	0.0023 mg/L	0.0023 mg/L	3:57:59 PM
3	Li 670.784	52052.6	47506.0	0.0023 mg/L	0.0023 mg/L	3:58:06 PM

Mean Data: DI water

Analyte	Mean Corrected Intensity	Calib. Conc. Units	Std.Dev.	Sample Conc. Units	Std.Dev.	RSD
Li 670.784	48038.7	0.0024 mg/L	0.00005	0.0024 mg/L	0.00005	2.12%

Replicate Data: sample A (dil x500)

Repl#	Analyte	Net Intensity	Corrected Intensity	Calib. Conc. Units	Sample Conc. Units	Analysis Time
1	Li 670.784	26196851.4	26192304.9	1.2907 mg/L	1.2907 mg/L	4:02:17 PM
2	Li 670.784	25743718.2	25739171.7	1.2684 mg/L	1.2684 mg/L	4:02:21 PM
3	Li 670.784	25737772.4	25733225.9	1.2681 mg/L	1.2681 mg/L	4:02:24 PM

Mean Data: sample A (dil x500)

Analyte	Mean Corrected Intensity	Calib. Conc. Units	Std.Dev.	Sample Conc. Units	Std.Dev.	RSD

Li 670.784	25888234.2	1.2757 mg/L	0.01298	1.2757 mg/L	0.01298	1.02%	

Lithium metal account: 1.2757 mg/ L * 500 * 1 mL = 0.64 mg
Lithium metal percentage in the grey powder: 42.6 %

2. Detection of K^+

The sample was collected from the following reaction.

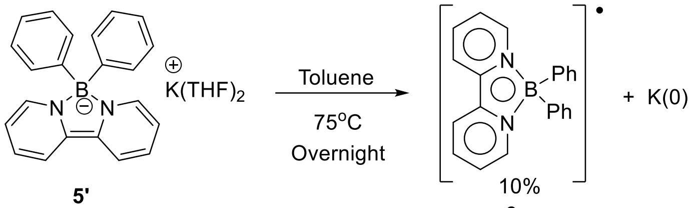

5'

6

Compound (**5’**) (35 mg, 0.070 mmol) was suspended in toluene (10 mL) at room temperature. The mixture was heated without stirring at 75°C overnight. The color of the toluene solution turned dark green. After filtration, the toluene solution was collected, and all the volatile was removed under a high vacuum, affording compound **6** (2.3 mg, 10%). The residue was then washed with THF (6 mL). The residue and funnel were quenched with 1 mL of deionized water and washed with 9 mL of deionized water. (collected 10 mL in total).

Replicate Data: DL

Repl#	Analyte	Net Intensity	Corrected Intensity	Calib. Conc. Units	Sample Analysis Conc. Units	Time
1	K 766.490	405069.8	395250.0	0.5903 mg/L	0.5903 mg/L	4:33:16 PM
2	K 766.490	400559.0	390739.3	0.5836 mg/L	0.5836 mg/L	4:33:20 PM
3	K 766.490	404626.6	394806.8	0.5897 mg/L	0.5897 mg/L	4:33:23 PM
4	K 766.490	409786.8	399967.0	0.5974 mg/L	0.5974 mg/L	4:33:26 PM
5	K 766.490	406449.5	396629.7	0.5924 mg/L	0.5924 mg/L	4:33:29 PM
6	K 766.490	396852.5	387032.7	0.5781 mg/L	0.5781 mg/L	4:33:31 PM
7	K 766.490	409078.5	399258.7	0.5963 mg/L	0.5963 mg/L	4:33:34 PM
8	K 766.490	406574.5	396754.7	0.5926 mg/L	0.5926 mg/L	4:33:37 PM
9	K 766.490	401793.2	391973.5	0.5855 mg/L	0.5855 mg/L	4:33:40 PM
10	K 766.490	403980.1	394160.3	0.5887 mg/L	0.5887 mg/L	4:33:43 PM

Mean Data: DL

Analyte	Mean Corrected Intensity	Calib. Conc. Units	Std.Dev.	Sample Conc. Units	Std.Dev.	RSD
K 766.490	394657.3	0.5895 mg/L	0.00587	0.5895 mg/L	0.00587	1.00%

Replicate Data: DI water

Repl#	Analyte	Net Intensity	Corrected Intensity	Calib. Conc. Units	Sample Analysis Conc. Units	Time
1	K 766.490	21176.9	11357.2	0.0170 mg/L	0.0170 mg/L	2:38:12 PM
2	K 766.490	20934.1	11114.3	0.0166 mg/L	0.0166 mg/L	2:38:18 PM
3	K 766.490	20786.0	10966.2	0.0164 mg/L	0.0164 mg/L	2:38:25 PM

Mean Data: DI water

Analyte	Mean Corrected Intensity	Calib. Conc. Units	Std.Dev.	Sample Conc. Units	Std.Dev.	RSD
K 766.490	11145.9	0.0166 mg/L	0.00029	0.0166 mg/L	0.00029	1.77%

Replicate Data: Sample A

Repl#	Analyte	Net Intensity	Corrected Intensity	Calib. Conc. Units	Sample Analysis Conc. Units	Time
1	K 766.490	6540856.8	6531037.0	9.7548 mg/L	9.7548 mg/L	2:40:45 PM
2	K 766.490	6539110.5	6529290.7	9.7522 mg/L	9.7522 mg/L	2:40:48 PM

3 K 766.490	6743331.4	6733511.6	10.057 mg/L	10.057 mg/L	2:40:50 PM

Mean Data: Sample A

Analyte	Mean Corrected Intensity	Calib. Conc. Units	Std.Dev.	Sample Conc. Units	Std.Dev.	RSD
K 766.490	6597946.5	9.8547 mg/L	0.17536	9.8547 mg/L	0.17536	1.78%

Potassium metal account: 9.8547 mg/ L * 10 * 1 mL = 0.099 mg

Crystallographic Details

Crystallographic details of **7·DCM**, **6** and **5**

	**7·DCM**	**6**	**5**
Empirical Formula	C23H20BCl4CuN2	C22H18BN2	C38H50BLiN2O4
Formula Weight	540.56	321.19	616.55
Temperature (K)	203(2)	193(2)	213(2)
Crystal System	Triclinic	Triclinic	Monoclinic
Space Group	P-1	P-1	P21
a (Å)	8.9711(8)	8.9948(3)	10.5613(3)
b (Å)	11.2052(10)	9.1299(4)	15.5054(5)
c (Å)	12.5573(10)	12.3211(5)	11.0474(4)
α (°)	100.954(3)	73.978(2)	90
β (°)	103.460(3)	78.431(2)	93.193(2)
γ (°)	95.983(3)	61.804(2)	90
Volume ($Å^3$)	1190.60(18)	854.18(6)	1806.28(10)
Z	2	2	2
ρcalc (g/cm^3)	1.508	1.249	1.134
Absorption Coefficient (mm^{-1})	1.380	0.073	0.560
F(000)	548	338	664
Crystal Size (mm^3)	0.32 x 0.21 x 0.03	0.33 x 0.15 x 0.04	0.25 x 0.15 x 0.03
Radiation/Å	0.71073	0.71073	0.71073
Θ range for data collection (°)	2.240 to 26.418	2.578 to 26.420	4.008 to 74.640
Index Ranges	-11<=h<=11<br>-13<=k<=13<br>-15<=l<=15	-11<=h<=11<br>-9<=k<=11<br>-15<=l<=15	-13<=h<=13<br>-19<=k<=18<br>-13<=l<=13
Reflections Collected	13299	6039	31596
Independent Reflections	4841 [R(int) = 0.0803]	3455 [R(int) = 0.0452]	7051 [R(int) = 0.0447]
Reflections with $I > 2\sigma(I)$	2860	2114	6535
Completeness / Θ full	0.994	0.982	0.999
Data/Restraints/Parameters	4841 / 0 / 283	3455 / 0 / 226	7051 / 967 / 559
Goodness-of-fit on F^2	1.022	1.016	1.055
Final R indexes [$I > 2\sigma(I)$]	R1 = 0.0699<br>wR2 = 0.1592	R1 = 0.0561<br>wR2 = 0.1139	R1 = 0.0471<br>wR2 = 0.1305
Final R indexes [all data]	R1 = 0.1253<br>wR2 = 0.1945	R1 = 0.1069<br>wR2 = 0.1466	R1 = 0.0496<br>wR2 = 0.1340
Largest diff. peak/hole (e $Å^{-3}$)	1.503 and -1.249	0.206 and -0.233	0.157 and -0.167
CCDC number	2301616	2301617	2301618

Computational Details

All calculations were carried out using ORCA (version 5.0.4).[9] Geometry optimization was performed at the density functional theory (DFT) using M062X [10] with a def2-TZVP [11] basis set. Counterions were omitted during calculations. The THF solution was modeled by the Conductor-like Polarizable Continuum Model (CPCM) [12] in optimization. Harmonic vibrational analyses were carried out to confirm if the optimized structure was a local minimum structure and to provide zero-point vibrational energy corrections and thermal corrections to various thermodynamic properties. Magnetic properties were calculated using revTPSS [13]/pcSseg-1 [14] level of theory with optimized structures with the SMD solvent model.[15] ACID was calculated from AICD2.0 software. [16]

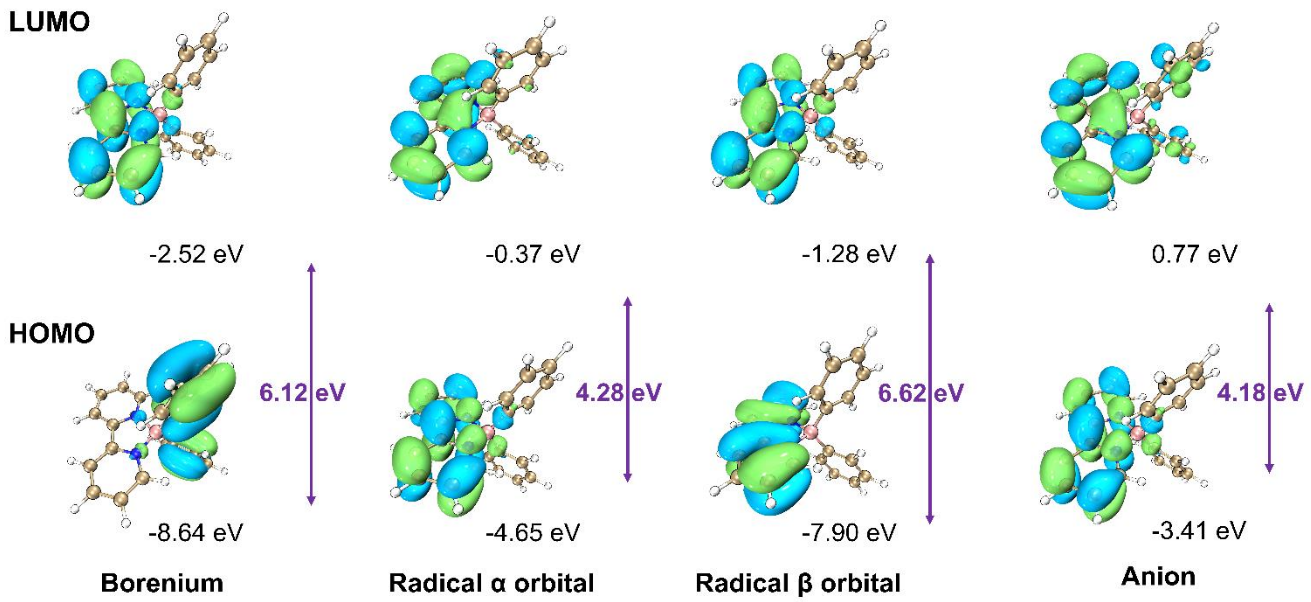

Supplementary Figure 41. Calculated LUMO and HOMO orbitals of boronium, boron radical, and boron anion (Isovalue: 0.03 a.u.).

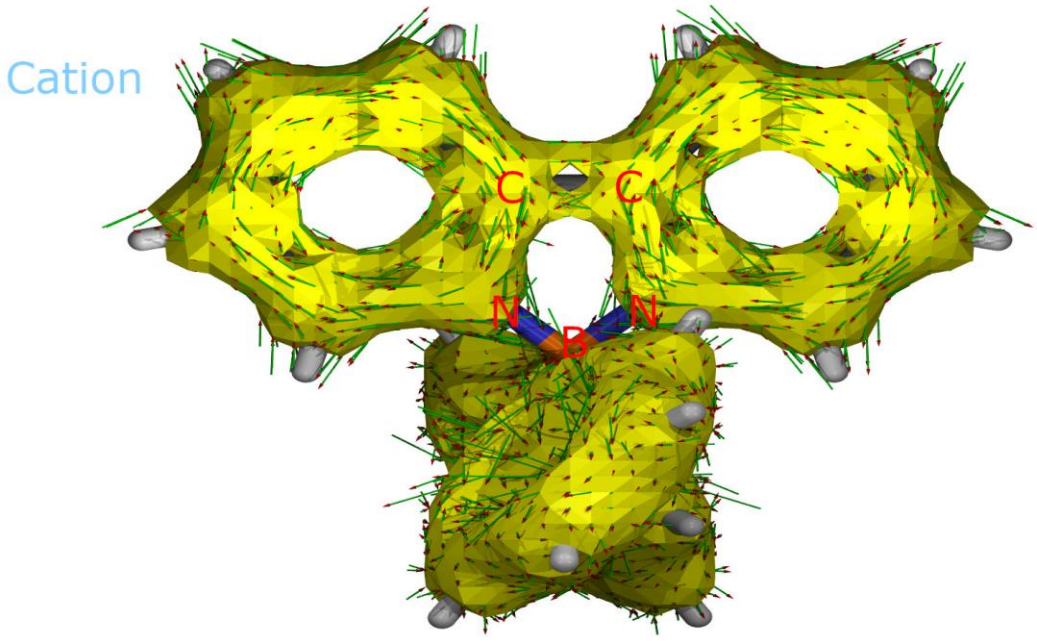

Supplementary Figure 42. AICD of boronium. The direction of magnetic field is perpendicular to the paper and point to the reader.

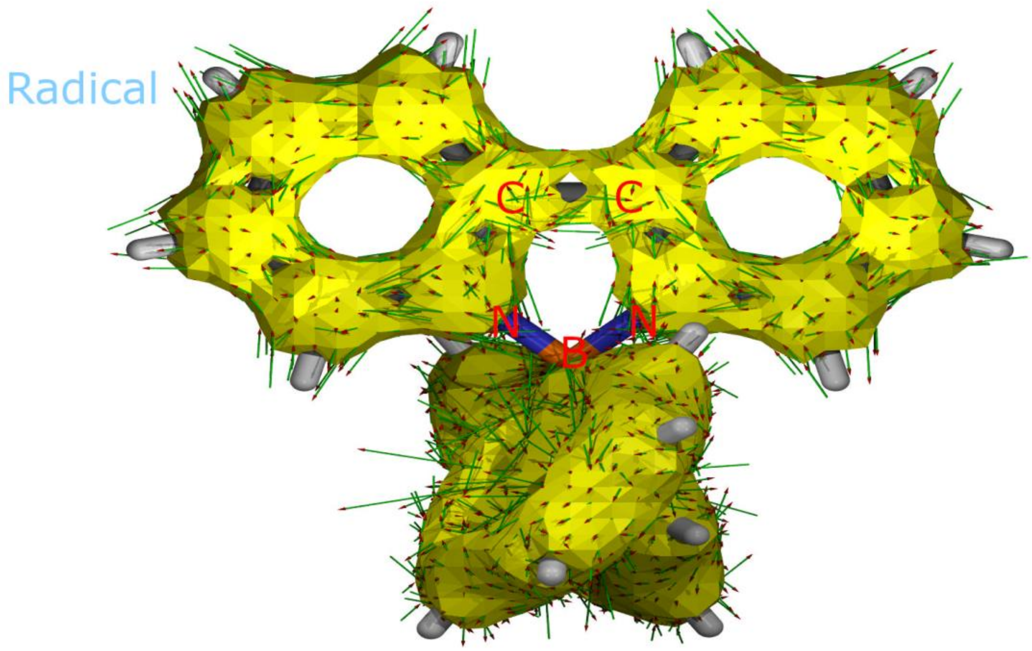

Supplementary Figure 43. AICD of radical. The direction of magnetic field is perpendicular to the paper and point to the reader.

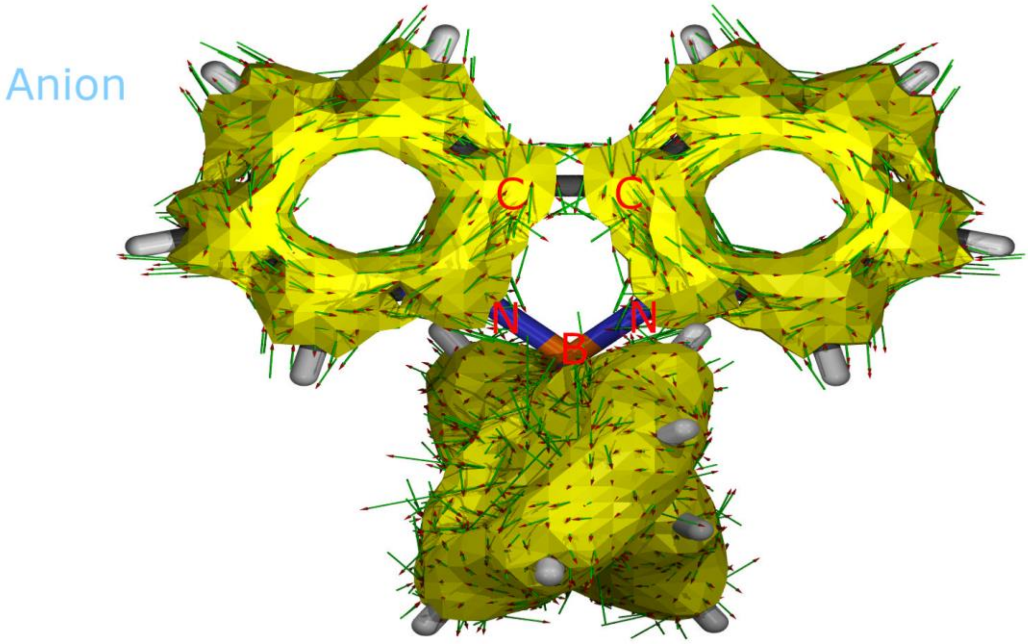

Supplementary Figure 44. AICD of anion. The direction of magnetic field is perpendicular to the paper and point to the reader.

A combination of computational and experimental methods was utilized to study the reduction of lithium.

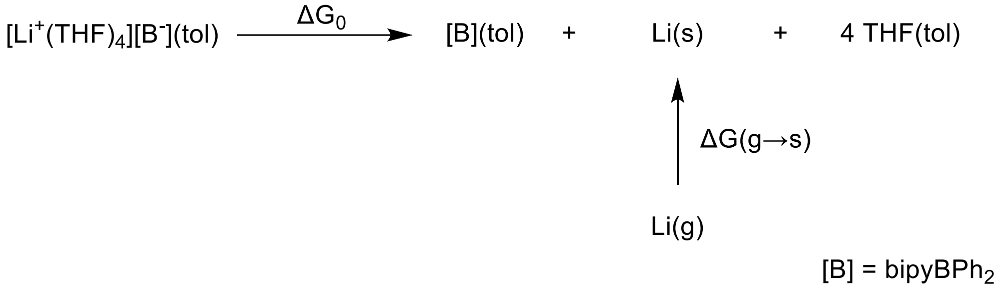

For the lithium decomposition reaction of compound **5**, total energy change could be obtained from the calculated Gibbs free energies, G{THF(tol)}, G{[B](tol)}, G{[Li+(THF)4][B-](tol)} and G{Li(s)}. To reduce the calculation error of a single atom, lithium gas to solid thermodynamic data were taken from the experimental results[17].

Geometry optimization was performed at the density functional theory (DFT) using M062X [10] with a def2-TZVP [11] basis set. Counterions were omitted during calculations. The toluene solution was modeled by the Conductor-like Polarizable Continuum Model (CPCM) [12] in optimization. Harmonic vibrational analyses were carried out to confirm if the optimized structure was a local minimum structure and to provide zero-point vibrational energy corrections and thermal corrections to various thermodynamic properties. For higher accuracy of electronic energy, double hybrid DFT, and larger basis set, revDSD-PBEB86-D4[18]/ma-def2-QZVP [11] level of theory with optimized structures was carried out with the SMD solvent model.[15]

5 #1	ΔG (kcal/mol) #2	ΔH (kcal/mol) #2
Single molecule	-26.21 (revDSD-PBEP86)	-17.78 (revDSD-PBEP86)
	1.24 (M06-2X)	-15.43 (M06-2X)
Complete ionization	-51.82 (revDSD-PBEP86)	-25.80 (revDSD-PBEP86)
	-31.21 (M06-2X)	-41.51 (M06-2X)

#1: It means how we considered the energy of Lithium borate anion in calculation. Single molecular means it was a single molecular ($[Li^+(THF)_4][B^-]$) in which the borate part and the lithium part were not ionized in toluene. Complete ionization means it was calculated in two parts, the borate part ($[B^-]$) and the lithium part ($[Li^+(THF)_4]$) in toluene.
#2: DFT method used for electronic energy calculations.

Detailed calculated energies for each molecule are listed below in Hartree(h).

Compound	G (M062X)	H(M062X)	G (revDSD-PBEP86)	H (revDSD-PBEP86)
THF(tol)	-232.3464071	-232.3129632	-231.4634135	-231.4299695
$[Li^+(THF)4][B^-](tol)$	-1920.182959	-1920.069231	-1912.6061219	-1912.492394
$[Li^+(THF)4](tol)$	-936.8466343	-936.7691602	-933.2591693	-933.1816953
[B](tol)	-983.2517569	-983.1863109	-979.2810932	-979.2156473
$[B^-](tol)$	-983.3496272	-983.2835549	-979.3389644	-979.272892
Li(g)	-7.495325	-7.4976869	-7.4648688	-7.4672293

And ΔH Li(g→s) = -159.3 kJ/mol, S Li(g) = 138.782 J/(mol*K) and S Li(s) = 29.12 J/(mol*K) were obtained from the experimental results.[17]

The ΔG revDSD-PBEP86 Single molecule in toluene can be obtained:
ΔG (kcal/mol) = G [B](tol) + G Li(g) + 4* G THF(tol) + ΔH Li(g→s) – (S Li(s) - S Li(g)) – G $[Li^+(THF)_4][B^-](tol)$
= -979.2810932h + (-7.4648688 h) + 4*(-231.4634135) h + -159.3/(627.51*4.18) h – 298.15 * (29.12-138.782)/(627.51*4.18*1000) h – (-1912.6061219 h)
= -0.041761 h = -26.21 kcal/mol
Similarly, ΔG revDSD-PBEP86 Complete ionization in toluene was calculated:
ΔG (kcal/mol) = G [B](tol) + G Li(g) + 4* G THF(tol) + ΔH Li(g→s) – (S Li(s) - S Li(g)) – G $[Li^+(THF)_4]$ (tol) -G $[B^-](tol)$
= -979.2810932h + (-7.4648688 h) + 4*(-231.4634135) h + -159.3/(627.51*4.18) h – 298.15 * (29.12-138.782)/(627.51*4.18*1000) h – (-933.2591693 h) - (-979.3389644 h)
= -0.049749 h = -31.21 kcal/mol

Reactions of Scheme 6-8 using naphthalene anion as reducing reagents

Sodium naphthalene was freshly prepared from a mixture of naphthalene and sodium in THF at -35°C (concentration was 0.1 mol/L).
For E-E reactions, only P-P was formed with a 21% conversion. The Sn-Sn, Se-Se, and Ge-Ge coupling products were not observed.
In the reductive coupling of pyridine, the formation of 4,4'-bipyridine was not observed.
In the Birch reduction of Acridine, no desired products were observed.

Sn-Sn coupling reactions:
To the C_6D_6 solution of *n*Bu_3SnCl (6.5 mg, 0.02 mmol, 1.0 eq.) in J. Young's tube was added 200 uL 0.1M Sodium naphthalene (0.02 mmol, 1.0eq.). The reaction was placed at room temperature overnight. No Sn-Sn products were observed based on the ^1H NMR.

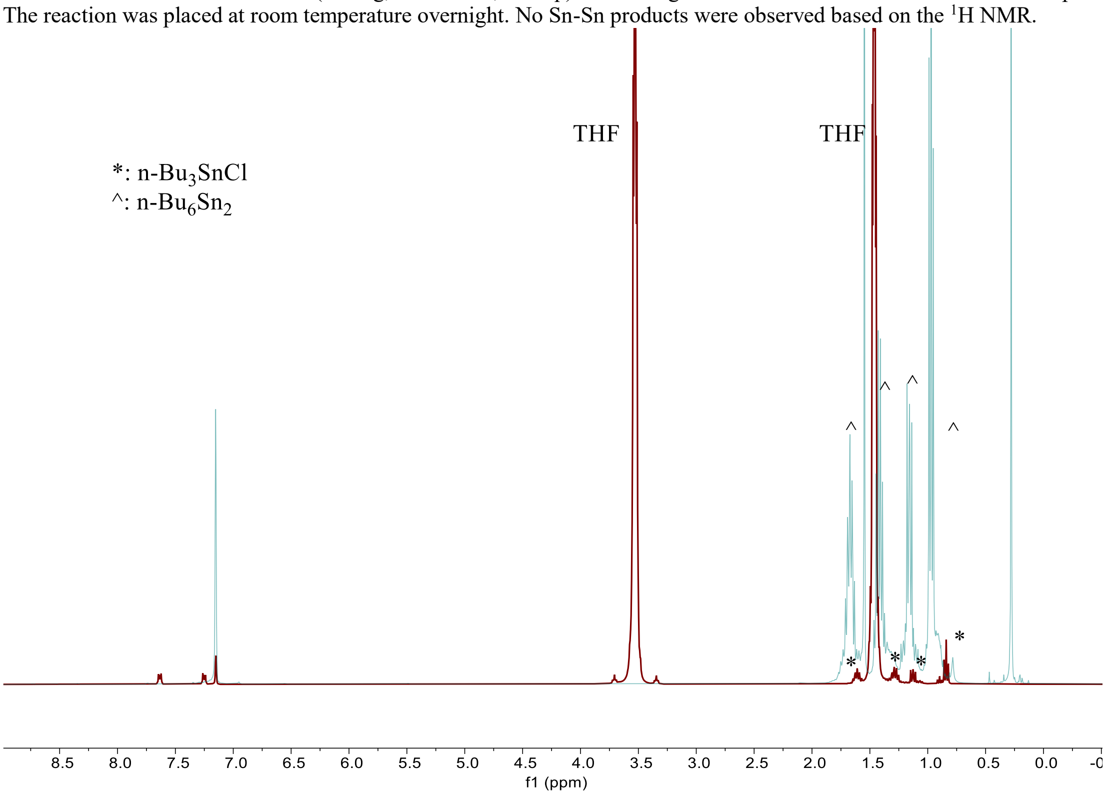

Supplementary Figure 45.^1H NMR in C_6D_6 for comparison (Sn-Sn). (Red: nBu_3SnCl + NaNap.; Green: nBu_6Sn_2).

Se-Se coupling reactions:

To the C_6D_6 solution of PhSeCl (3.8 mg, 0.02 mmol, 1.0 eq.) in J. Young's tube was added 200 uL 0.1M Sodium naphthalene (0.02 mmol, 1.0eq.). The reaction was placed at room temperature overnight. No generation of Se-Se compounds was observed based on the ^{1}H NMR.

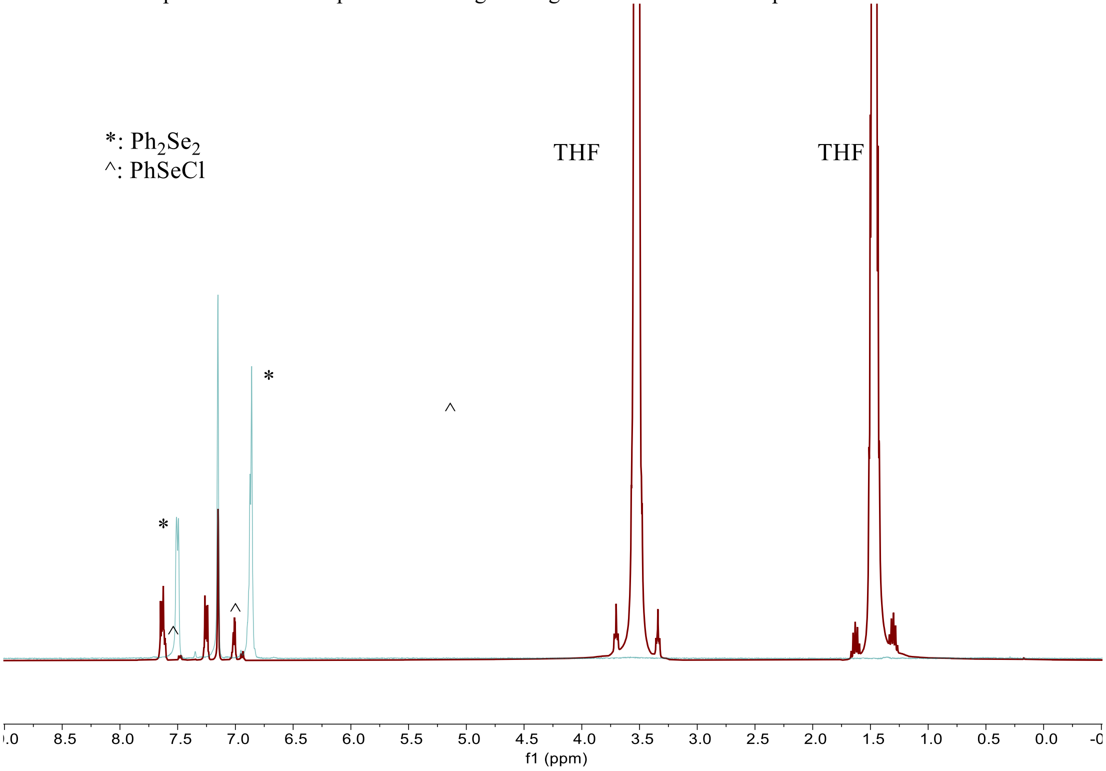

Supplementary Figure 46. ^{1}H NMR in C_6D_6 for comparison (Ge-Ge). (Red: PhSeCl+ NaNap.; Green: Ph_2Se_2).

Ge-Ge coupling reactions:

To the C_6D_6 solution of Et_3GeCl (3.9 mg, 0.02 mmol, 1.0 eq.) in J. Young's tube was added 200 uL 0.1M Sodium naphthalene (0.02 mmol, 1.0eq.). The reaction was placed at room temperature overnight. No generation of Ge-Ge compounds was observed based on the ^1H NMR.

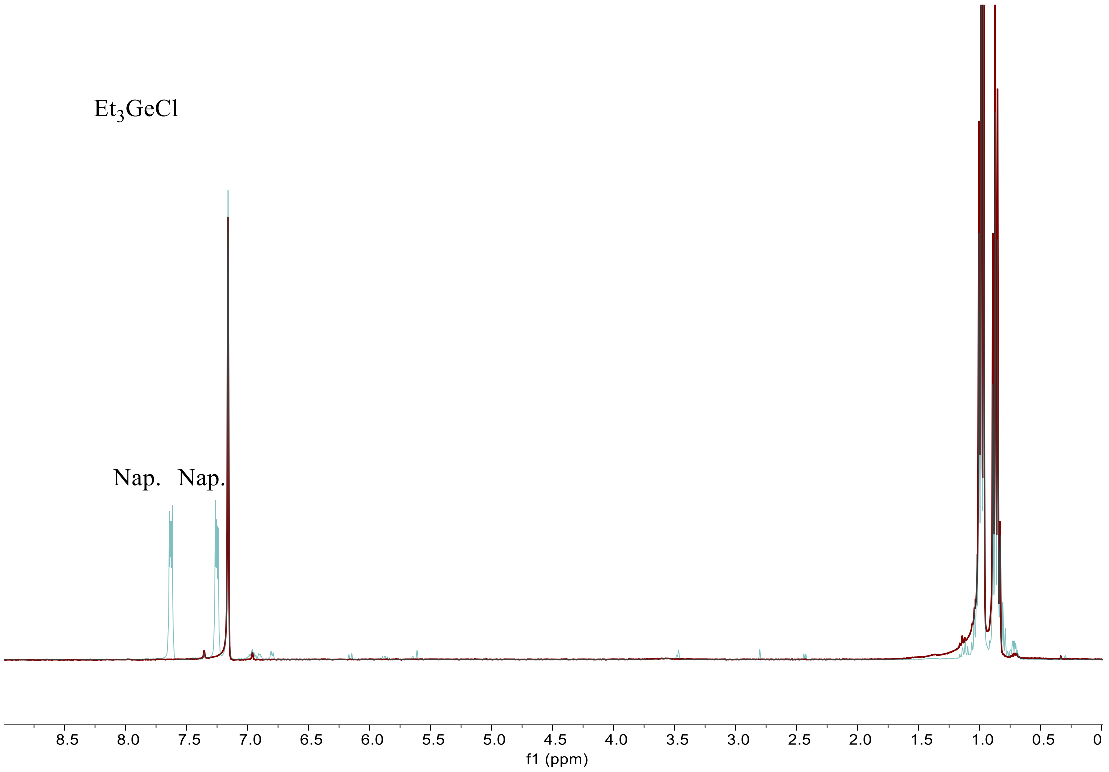

Supplementary Figure 47. ^1H NMR in C_6D_6 for comparison (Ge-Ge). (Red: Et_3GeCl; Green: Et_3GeCl+ NaNap., removal of solvent).

P-P coupling reactions:

To the C_6D_6 solution of Ph_2PCl (4.4 mg, 0.02 mmol, 1.0 eq.) in J. Young's tube was added 200 uL 0.1M Sodium naphthalene (0.02 mmol, 1.0eq.). The reaction was placed at room temperature overnight. About 21% of P-P compounds were generated based on the ^{31}P NMR.

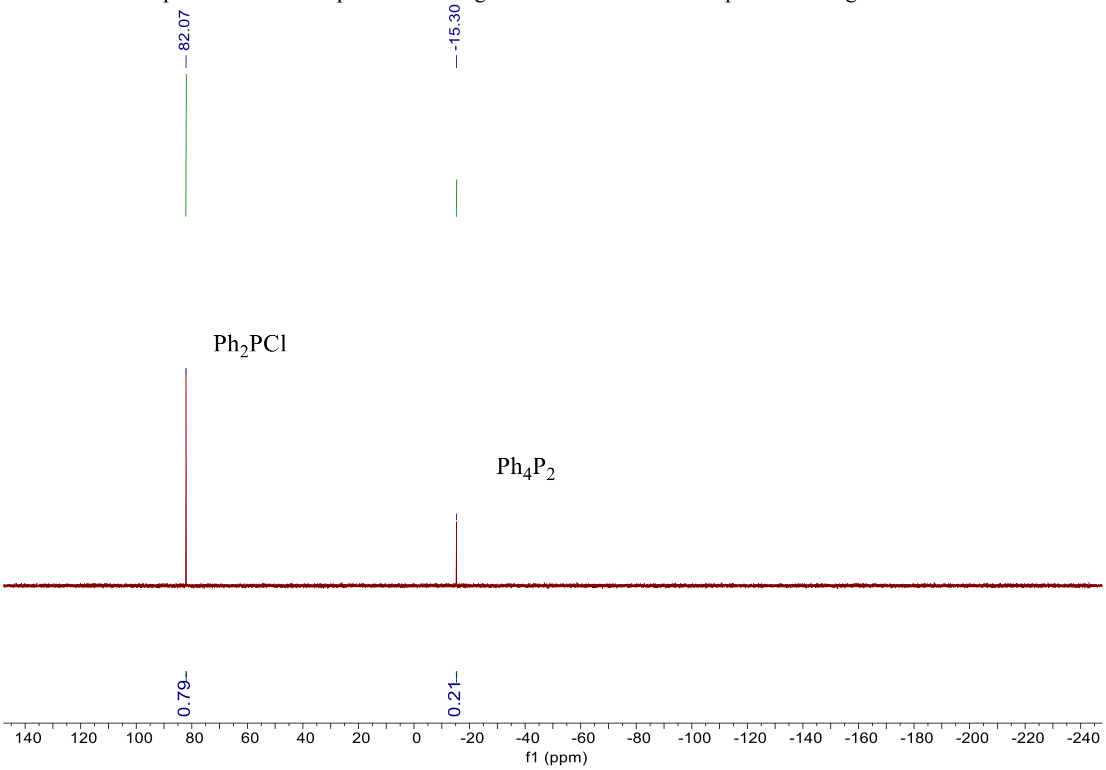

Supplementary Figure 48. $^{31}P\{^1H\}$ NMR in C_6D_6 for comparison (P-P).

Reductive Pyridine couplings:
To 0.1M Sodium naphthalene (0.63 mmol, 1.0 eq.), pyridine (50 mg, 0.63 mmol, 1.0 eq.) was added. After stirring at room temperature overnight. drops of MeOH were added to quench the reaction. No 4,4'-bipyridine was observed based on TLC (EA: DCM=2:1, 1%NEt_3).

The reduction of acridine:
To 0.1M Sodium naphthalene (0.28 mmol, 1.0 eq.), acridine (50 mg, 0.28 mmol, 1.0 eq.) was added. After stirring at room temperature overnight. drops of MeOH were added to quench the reaction. The reduction product was not observed on TLC (EA: Hex=1:5).

References

[1] Fulmer,. G. R., Miller, A. J. M., Sherden, N. H., Gottlieb, H. E., Nudelman, A., Stoltz, B. M., Bercaw, J. E., Goldberg, K. I. NMR Chemical Shifts of Trace Impurities: Common Laboratory Solvents, Organics, and Gases in Deuterated Solvents Relevant to the Organometallic Chemist. *Organometallics* ***29***, 2176-2179 (2010).

[2] Dodds, D. L., Haddow, M. F., Orpen, A. G., Pringle, P. G., Woodward, G. Stereospecific Diphosphination of Activated Acetylenes: A General Route to Backbone-Functionalized, Chelating 1,2-Diphosphinoethenes *Organometallics* ***25***, 5937–5945 (2006).

[3] Elangovan, S., Irran, E., Klare, H. F. T., Oestreich, M. Cationic Cobalt–Thiolate Complexes for the Dehydrogenative Coupling of nBu$_3$SnH. *Organometallics* ***41***, 852-857 (2022).

[4] Leng, T., Wu, G., Zhou, Y. B., Gao, W., Ding, J., Huang, X., Liu, M., Wu, H. Silver-Catalyzed One-Pot Three-Component Selective Synthesis of β-Hydroxy Selenides. *Adv. Synth. Catal.* ***360***, 4336-4340 (2018).

[5] Guan, W. Lu, L., Jiang, Q., Gittens, A. F., Wang, Y., Novaes, L. F. T., Klausen, R. S., Lin, S. An Electrochemical Strategy to Synthesize Disilanes and Oligosilanes from Chlorosilanes. *Angew. Chem. Int. Ed*. ***62***, e202303592 (2023).

[6] Davison, N., Quirk, J. A., Tuna, F., Collison, D., McMullin, C. L., Michaels, H., Morritt, G. H., Waddell, P. G., Gould, J. A., Freitag, M., Dawson, J. A., Lu, E. A room-temperature-stable electride and its reactivity: Reductive benzene/pyridine couplings and solvent-free Birch reductions. *Chem* ***9***, 576-591 (2023).

[7] Stoll, S., Schweiger, A. EasySpin, a comprehensive software package for spectral simulation and analysis in EPR. *J. Magn. Reson.* ***178***, 42-55 (2006).

[8] Trivedi, M. K., Tallapragada, R. M., Branton, A., Trivedi, D., Nayak, G., Latiyal, O., Jana, S. Physical, Atomic and Thermal Properties of Biofield Treated Lithium Powder. *J. Adv. Chem. Eng.* ***5***, hal-01491589 (2015).

[9] a) Neese, F. The ORCA program system. *Wiley Interdiscip. Rev.: Comput. Mol.* ***2***, 73-78 (2011); b) Neese, F. Software update: The ORCA program system—Version 5.0. *Wiley Interdiscip. Rev.: Comput. Mol.* ***12***, e1606 (2022).

[10] Zhao, Y. & Truhlar, D. G. The M06 suite of density functionals for main group thermochemistry, thermochemical kinetics, noncovalent interactions, excited states, and transition elements: two new functionals and systematic testing of four M06-class functionals and 12 other functionals. *Theor. Chem. Acc.* ***120***, 215-241 (2008).

[11] Weigend, F. & Ahlrichs, R. Balanced basis sets of split valence, triple zeta valence and quadruple zeta valence quality for H to Rn: Design and assessment of accuracy. *Phys. Chem. Chem. Phys.* ***7***, 3297-3305 (2005).

[12] Barone, V. & Cossi, M. Quantum Calculation of Molecular Energies and Energy Gradients in Solution by a Conductor Solvent Model. *J. Phys. Chem. A* ***102***, 1995-2001 (1998).

[13] Perdew, J. P., Ruzsinszky, A., Csonka, G. I., Constantin, L. A., Sun, J. Workhorse Semilocal Density Functional for Condensed Matter Physics and Quantum Chemistry. *Phys. Rev. Lett. 103*, 026403 (2009).

[14] Jensen, F. Segmented Contracted Basis Sets Optimized for Nuclear Magnetic Shielding. *J. Chem. Theory Comput.* ***11***, 132-138 (2015).

[15] Marenich, A. V., Cramer, C. J., Truhlar, D. G. Universal Solvation Model Based on Solute Electron Density and on a Continuum Model of the Solvent Defined by the Bulk Dielectric Constant and Atomic Surface Tensions. *J. Phys. Chem. B* ***113***, 6378-6396 (2009).

[16] Geuenich, D., Hess, K., Köhler, K., Herges, R. Anisotropy of the Induced Current Density (ACID), a General Method To Quantify and Visualize Electronic Delocalization. *Chem. Rev.* ***105***, 3758-3772 (2005).

[17] Cox, J. D., Wagman, D. D., and Medvedev, V. A., CODATA Key Values for Thermodynamics, Hemisphere Publishing Corp., New York, 1989.

[18] (a) Santra, G., Sylvetsky, N., Martin, J. M. L., Minimally Empirical Double-Hybrid Functionals Trained against the GMTKN55 Database: revDSD-PBEP86-D4, revDOD-PBE-D4, and DOD-SCAN-D4. *J. Phys. Chem. A* ***123***, 5129-5143 (2019) (b) Caldeweyher, E., Bannwarth, C., Grimme, S. Extension of the D3 dispersion coefficient model. *J. Chem. Phys.* ***147***, 034112 (2017). (c) Caldeweyher, E., Ehlert, S., Hansen, A., Neugebauer, H., Spicher, S., Bannwarth, C., Grimme, S. A generally applicable atomic-charge dependent London dispersion correction. *J. Chem. Phys.* ***150***, 154122 (2019).
